# Characterization of evolutionarily distinct rice *BAHD‐Acyltransferases* provides insight into their plausible role in rice susceptibility to *Rhizoctonia solani*

**DOI:** 10.1002/tpg2.20140

**Published:** 2021-09-09

**Authors:** Gulshan Kumar, Pankaj Kumar, Ritu Kapoor, Jagjeet Singh Lore, Dharminder Bhatia, Arun Kumar

**Affiliations:** ^1^ Biotechnology Division CSIR‐Institute of Himalayan Bioresource Technology Palampur Himachal Pradesh 176061 India; ^2^ National Agri‐Food Biotechnology Institute Mohali Punjab 140306 India; ^3^ Department of Plant Breeding and Genetics Punjab Agricultural University Ludhiana 141 004 India; ^4^ School of Agricultural Biotechnology Punjab Agricultural University Ludhiana 141004 India; ^5^ Academy of Scientific and Innovative Research Ghaziabad Uttar Pradesh 201002 India

## Abstract

Plants produce diverse secondary metabolites in response to different environmental cues including pathogens. The modification of secondary metabolites, including acylation, modulates their biological activity, stability, transport, and localization. A plant‐specific *BAHD‐acyltransferase* (*BAHD‐AT*) gene family members catalyze the acylation of secondary metabolites. Here we characterized the rice (*Oryza sativa* L.) *BAHD‐ATs* at the genome‐wide level and endeavor to define their plausible role in the tolerance against *Rhizoctonia solani* AG1‐IA. We identified a total of 85 rice *OsBAHD‐AT* genes and classified them into five canonical clades based on their phylogenetic relationship with characterized BAHD‐ATs from other plant species. The time‐course RNA sequencing (RNA‐seq) analysis of *OsBAHD‐AT* genes and qualitative real‐time polymerase chain reaction (qRT‐PCR) validation showed higher expression in sheath blight susceptible rice genotype. Furthermore, the DNA methylation analysis revealed higher hypomethylation of *OsBAHD‐AT* genes that corresponds to their higher expression in susceptible rice genotype, indicating epigenetic regulation of *OsBAHD‐AT* genes in response to *R. solani* AG1‐IA inoculation. The results shown here indicate that BAHD‐ATs may have a negative role in rice tolerance against *R. solani* AG1‐IA possibly mediated through the brassinosteroid (BR) signaling pathway. Altogether, the present analysis suggests the putative functions of several *OsBAHD‐AT* genes, which will provide a blueprint for their functional characterization and to understand the rice–*R. solani* AG1‐IA interaction.

## Core Ideas

- *OsBAHD‐AT* genes involved in acylation of secondary metabolites identified in the rice genome.
- *OsBAHD‐AT* genes were differentially expressed in response to *R. solani* AG1‐IA infection.
- Higher expression of *OsBAHD‐AT* genes in susceptible genotype suggested role in susceptibility.
- The epigenetic regulation via cytosine DNA methylation of *OsBAHD‐AT* genes was revealed.

**Abbreviations**
BAHD‐AT: BAHD‐acyltransferase
BR: brassinosteroid
cDNA: complementary DNA
DMR: differential methylated region
hpi: hours postinoculation
LG: linkage group
qRT‐PCR: qualitative real‐time polymerase chain reaction
QTL: quantitative trait loci
RNA‐seq: RNA sequencing
SA: salicylic acid.

## 1 INTRODUCTION

Plant secondary metabolites play an important role in diverse processes involved in growth and development and resistance to various biotic and abiotic stresses (Erb & Kliebenstein, 2020). Although a few pathways are involved in the biosynthesis of major classes of plant secondary metabolites, the modifications and the expansion of the basic skeleton result in the immense diversity of the plant's secondary metabolites (Wang et al., 2019). These modifications and expansion processes involve various biochemical reactions such as decarboxylation, hydroxylation, glycosylation, methylation, oxidation and reduction, and acylation (Wang et al., 2019). Among these biochemical reactions, acylation is the most prevalent modification that expands the diversity of phenolic compounds synthesized through various pathways (Bontpart et al., 2015). The acylation of the metabolites influences their properties, functions, transport, and storage. For instance, acylation of anthocyanins modulate their solubilization, stability and localization, and color properties (Gomez et al., 2009; Mugford et al., 2009; Nakayama et al., 2003).

The acylation is catalyzed by acyltransferases, where activated acyl donor, such as acyl‐sugars, acylated‐proteins, or acylated‐CoA thioesters, donate their acyl group to modify the secondary metabolites (Bontpart et al., 2015). The acyltransferases belong to the large protein family and are broadly categorized into two major classes based on their specificity for the activated acyl donors: BAHD‐ATs responsible for acyl‐CoA dependent acylation, while the Serine carboxypeptidase‐like‐acyltransferases are responsible for 1‐O‐β‐glucose esters dependent acylation (Bontpart et al., 2015).

The BAHD‐AT name is derived from the first four characterized enzymes of the family (namely, benzylalcohol O‐acetyltransferase, BEAT; anthocyanin O‐hydroxycinnamoyltransferase, AHCT; anthranilate N‐hydroxycinnamoyl/benzoyltransferase, HCBT; and deacetylvindoline 4‐O‐acetyltransferase, DAT) (Dudareva et al., 1998; Fujiwara et al., 1998; St‐Pierre et al., 1998; Yang et al., 1997). All the biochemically characterized BAHD‐ATs, hitherto, are of molecular weight ranging from 48 to 55 kDa and known to function as monomeric enzymes (Bontpart et al., 2015; D'Auria, 2006). Though the sequence similarity among the members of the BAHD‐ATs family is only 25–34% at protein level (Ma et al., 2005), members with functional similarity may share the sequence identity of up to 90% (Boatright et al., 2004). Despite low sequence similarity, the BAHD‐AT members share two motifs: a conserved HXXXD(G) motif involved in substrate binding and catalysis and a less conserved DFGWG motif with a structural role in the catalytic activity of BAHD‐ATs (Unno et al., 2007). The HXXXD(G) motif of BAHD‐ATs is essential for the catalytic activity as determined using site‐directed mutagenesis (Bayer et al., 2004).

All BAHD‐ATs are plant‐specific and the members of this family have some unique role in plants. For example, Peng et al. (2016) showed the involvement of rice (*Oryza sativa* L.) BAHD‐ATs in the synthesis of an aromatic amine conjugate named phenolamides, which are hydroxycinnamoyl acylated products of the phenolic acid. The natural diversity in the phenolamides is derived from the allelic variation of the *BAHD‐AT* genes responsible for their biosynthesis. A member of the rice *BAHD‐AT* genes fmaily, OsAT1, imparted resistance responses against bacterial blight and blast diseases in rice (Mori et al., 2007). The rice line overexpressing *OsAT10* exhibited an alteration in the content of ferulic acid and *para*‐coumaric acid (hydroxycinnamic acids) linked to the cell wall polysaccharide matrix and led to the enhanced saccharification (20–40%) (Bartley et al., 2013). The plant BAHD family enzymes have complex substrates (a variety of acyl‐donors and acceptors) and different products. It is very difficult to predict the substrate specificity of uncharacterized BAHD family enzymes because (a) of the minimal sequence similarity among the members of *BAHD* gene family and (b) the multisite acylation pathways of BAHD‐family acyltransferases are poorly understood (Bontpart et al., 2015; Chiang et al., 2018; Wang et al., 2020). The first *BAHD* gene to be functionally identified was *glossy2* (*Zea mays* L.)*/CER2* [*Arabidopsis thaliana* (L.) Heynh.], which was associated with extending epicuticular waxes to limit water loss and protect against pathogenic invasion (D'Auria, 2006). Recently, Xu et al. (2021) identified and characterized the expression of the BAHD family during development, ripening, and stress response in banana (*Musa acuminata* Colla). These studies suggest the diverse roles of BAHD‐ATs and offer opportunities to explore their new roles and functions.

Sheath blight is an emerging and destructive disease in rice, which is caused by a soil‐borne fungal pathogen *Rhizoctonia solani* AG1‐IA. It is a necrotrophic fungal pathogen with a broad host range that can infect plant species from 32 taxonomic groups (Molla et al., 2020). The lack of significant resistance against sheath blight in wild and cultivated rice species limits the conventional plant breeding or genetic techniques to impart sheath blight resistance in rice. Sheath blight appears to be a quantitative trait that is controlled by the combinatorial action of several genes with minor effects (Singh et al., 2019). Though several minor quantitative trait loci (QTL) for resistance to sheath blight were mapped in rice, their validity is still not confirmed, and no major resistant gene has been identified to date (Channamallikarjuna et al., 2010; Yuan et al., 2019). In addition, several factors, including plant architecture and environmental conditions, also influence the disease phenotype (Jia et al., 2013) and therefore, confound the trait dissection. Identification of susceptibility genes could offer a promising solution to engineer rice for enhanced tolerance to sheath blight, but a lack of thorough understanding of mechanisms of rice–*R. solani* AG1‐IA interaction at the molecular level makes this task difficult. The recent advancement in the genomic tools, RNA‐sequencing (RNA‐seq) and methylome analysis, helps to provide various datasets that can be comprehensively integrated to develop the functional footprint of phenotype and the underlying mechanisms of host–pathogen interactions (Karre et al., 2017, 2019; A. Kumar et al., 2016). Owing to the diverse role of the *BAHD‐AT* gene family in plants, here, we performed genome‐wide identification, comparative phylogenetic analysis, and expression profiling of rice *BAHD‐ATs* in response to an Indian isolate of *R. solani* AG1‐IA in two rice genotypes with varying level of tolerance to sheath blight. In addition to using whole‐genome bisulfite sequencing, we also performed the cytosine DNA methylation profiling of rice *BAHD‐AT* genes. Based on the expression profiling, the putative candidate genes were identified and their plausible role in rice–*R. solani* AG1‐IA interaction is discussed.

## 2 MATERIALS AND METHODS

### 2.1 Data retrieval

The complete rice genome and the protein sequence data sets were downloaded from Rice Genome Annotation Project (http://rice.plantbiology.msu.edu) and The Rice Annotation Project (https://rapdb.dna.affrc.go.jp) to identify and annotate the rice *BAHD* gene family members. Similarly, the protein sequences of other plant species, that is*, Arabidopsis* were retrieved from TAIR10 (http://www. arabidopsis.org), while soybean [*Glycine max* (L.) Merr.], sorghum [*Sorghum bicolor* (L.) Moench], maize (*Zea mays* L.), and *Brachypodium distachyon* (L.) Beauv. protein sequences were retrieved from Ensemble plants (https://plants.ensembl.org/index.html).

### 2.2 Identification of BAHD‐ATs gene family

We retrieved the Hidden Markov Model profile of transferase domain (PF02458.15) from Pfam v31.0 and used it to identify the putative BAHD‐AT proteins in rice, *Arabidopsis*, soybean, sorghum, maize, and *B. distachyon* protein sequence datasets. For the identification, the ‘hmmsearch’ of HMMER V3.2 was performed, with a cut‐off e‐value 1 × 10^−4^. The presence of transferase domain, as well as other domains, in the identified putative BAHD proteins was determined by using the PfamScan program (parameters: ‐e_seq 1e^−04^ ‐e_dom 1e^−04^ ‐clan_overlap). The putative BAHD proteins were then further subjected to ‘find individual motif occurrences’ search for the presence of two signature motifs—HXXXDG and DFGWG—with an e‐value of 1 × 10^−3^. Parallelly, we also used the genetically or biochemically characterized BAHD‐AT sequences from the previously published data (D'Auria, 2006) for local BLASTp search against the rice protein dataset to identify any left‐out rice BAHD‐AT member.

**Core Ideas**

- *OsBAHD‐AT* genes involved in acylation of secondary metabolites identified in the rice genome.
- *OsBAHD‐AT* genes differentially expressed in their response to *R. solani* AG1‐IA infection.
- Higher expression of *OsBAHD‐AT* genes in susceptible genotype suggested role in susceptibility.
- The epigenetic regulation via cytosine DNA methylation of *OsBAHD‐AT* genes was revealed.

### 2.3 Chromosomal mapping, nomenclature, gene duplication, structural analysis, and in silico characterization of rice BAHD‐ATs

We retrieved a general feature format (gff) file from The Rice Annotation Project to extract the genomic coordinates of rice BAHD‐AT encoding genes. The extracted genomic coordinates were then mapped on the 12 rice chromosomes using the MapInspect program (http://mapinspect.apponic.com/). Since the NCBI and other databases have obscure nomenclature of rice BAHD‐AT proteins, the identified rice BAHD‐AT proteins were named by adding the suffix ‘*OsBAHD*’ to designate rice BAHD‐AT protein and numbered according to the genomic coordinates of the corresponding gene model from top to bottom on 1 to 12 rice chromosomes (Table 1). For the duplication analysis of the rice *BAHD‐AT* family, the result of all‐vs.‐all BLASTp analysis of total rice protein sequences was used to identify the collinear pairs of *BAHD‐AT* genes using the MCScanX tool with default parameters (Y. Wang et al., 2012). The extracted genomic coordinates were also used to schematically represent the intron–exon structure of *OsBAHD‐ATs* using Gene Structure Display Server (gsds.cbi.pku.edu.cn/). The subcellular localization of *OsBAHD‐AT* gene family members was predicted by using web servers, CELLO v.2.5 (Yu et al., 2006) and WoLF PSORT (Horton et al., 2007).

**TABLE 1:** The list of OsBAHD‐ATs and their chromosomal location and the characteristics of encoded proteins

Name	MSU locus ID	Chromosome	Strand	Position (start)	Position (end)	Length	Size	Isoelectric point
				————bp (5′–3′)————		(aa)^a^	kDa	pI
*OsBAHD01*	LOC_Os01g01520.1	Chr1	–	270,145	275,084	469	51.27	7.01
*OsBAHD02*	LOC_Os01g08380.1	Chr1	–	4,110,380	4,112,127	438	46.86	5.65
*OsBAHD03*	LOC_Os01g09010.1	Chr1	+	4,527,986	4,532,893	420	46.17	5.72
*OsBAHD04*	LOC_Os01g15709.1	Chr1	+	8,835,574	8,837,002	461	48.76	8.1
*OsBAHD05*	LOC_Os01g18620.1	Chr1	–	10,488,948	10,490,994	623	66.67	6.83
*OsBAHD06*	LOC_Os01g18744.1	Chr1	–	10,561,328	10,565,325	440	46.89	5.81
*OsBAHD07*	LOC_Os01g42870.1	Chr1	–	24,395,057	24,396,820	447	46.5	4.73
*OsBAHD08*	LOC_Os01g42880.1	Chr1	–	24,397,508	24,400,705	425	45.71	4.89
*OsBAHD09*	LOC_Os01g63480.1	Chr1	+	36,794,890	36,797,406	484	51.63	8.29
*OsBAHD10*	LOC_Os02g28170.1	Chr2	–	16,678,144	16,680,057	489	52.42	6.83
*OsBAHD11*	LOC_Os02g28200.1	Chr2	–	16,691,918	16,693,524	506	53.81	8.54
*OsBAHD12*	LOC_Os02g28220.1	Chr2	+	16,702,131	16,703,949	435	48.44	11.76
*OsBAHD13*	LOC_Os02g28300.1	Chr2	+	16,726,375	16,727,995	475	50.84	5.61
*OsBAHD14*	LOC_Os02g28340.1	Chr2	+	16,750,077	16,751,771	468	50.12	6.38
*OsBAHD15*	LOC_Os02g28410.1	Chr2	+	16,805,941	16,807,323	461	48.67	7.04
*OsBAHD16*	LOC_Os02g28470.1	Chr2	+	16,835,055	16,836,519	335	35.87	5.88
*OsBAHD17*	LOC_Os02g39850.1	Chr2	+	24,080,704	24,084,764	443	47.95	6.51
*OsBAHD18*	LOC_Os03g08720.1	Chr3	–	4,492,084	4,494,120	457	48.33	6.29
*OsBAHD19*	LOC_Os03g45740.1	Chr3	+	25,832,041	25,833,820	457	50.96	7.44
*OsBAHD20*	LOC_Os03g47860.1	Chr3	+	27,208,712	27,210,100	463	50.1	6.96
*OsBAHD21*	LOC_Os04g09590.1	Chr4	–	5,152,089	5,153,384	432	45.38	5.47
*OsBAHD22*	LOC_Os04g11810.1	Chr4	+	6,471,799	6,473,124	442	46.09	4.9
*OsBAHD23*	LOC_Os04g12820.1	Chr4	–	7,082,093	7,083,520	323	33.48	11.81
*OsBAHD24*	LOC_Os04g30570.1	Chr4	–	18,258,071	18,259,484	446	47.97	6.93
*OsBAHD25*	LOC_Os04g42250.2	Chr4	+	24,993,831	24,999,600	443	48.5	6.25
*OsBAHD26*	LOC_Os04g42250.3	Chr4	+	24,993,831	24,999,534	443	48.5	6.25
*OsBAHD27*	LOC_Os04g42250.4	Chr4	+	24,993,910	24,999,600	443	48.5	6.25
*OsBAHD28*	LOC_Os04g51660.1	Chr4	–	30,608,765	30,610,406	467	49.82	5.76
*OsBAHD29*	LOC_Os04g54560.1	Chr4	–	32,448,490	32,450,325	445	47.92	6.94
*OsBAHD30*	LOC_Os04g54570.1	Chr4	–	32,453,265	32,455,214	413	44.24	6.19
*OsBAHD31*	LOC_Os04g56910.1	Chr4	–	33,928,678	33,930,683	450	48.61	5.26
*OsBAHD32*	LOC_Os05g04584.1	Chr5	+	2,143,789	2,150,279	437	46.65	5.28
*OsBAHD33*	LOC_Os05g08640.1	Chr5	–	4,735,050	4,737,049	440	47.17	6.78
*OsBAHD34*	LOC_Os05g19910.1	Chr5	–	11,609,440	11,615,022	433	47.37	6.35
*OsBAHD35*	LOC_Os05g22300.1	Chr5	+	12,621,464	12,622,964	465	51.22	8.85
*OsBAHD36*	LOC_Os05g25150.1	Chr5	+	14,572,927	14,574,360	478	52.06	6.67
*OsBAHD37*	LOC_Os05g37660.1	Chr5	–	22,040,054	22,041,963	481	51.48	8.64
*OsBAHD38*	LOC_Os06g01350.1	Chr6	+	212,913	214,728	462	49.85	6.71
*OsBAHD39*	LOC_Os06g05284.1	Chr6	+	2,380,595	2,382,937	491	52.58	6.28
*OsBAHD40*	LOC_Os06g05300.1	Chr6	+	2,384,313	2,386,240	490	52.13	5.2
*OsBAHD41*	LOC_Os06g05310.1	Chr6	+	2,387,398	2,390,052	449	48.31	7.18
*OsBAHD42*	LOC_Os06g05320.1	Chr6	+	2,394,821	2,396,562	486	51.65	5.99
*OsBAHD43*	LOC_Os06g05750.1	Chr6	+	2,592,555	2,594,478	475	50.44	8.72
*OsBAHD44*	LOC_Os06g05790.1	Chr6	–	2,631,869	2,635,262	601	64.12	8.13
*OsBAHD45*	LOC_Os06g06180.1	Chr6	–	2,861,700	2,863,179	464	49.85	6.43
*OsBAHD46*	LOC_Os06g08580.1	Chr6	+	4,268,969	4,270,771	446	47.21	7.81
*OsBAHD47*	LOC_Os06g08640.1	Chr6	–	4,301,557	4,303,121	434	45.69	6.59
*OsBAHD48*	LOC_Os06g39390.1	Chr6	–	23,379,156	23,381,415	444	47.39	6.18
*OsBAHD49*	LOC_Os06g39470.1	Chr6	+	23,438,875	23,440,545	432	46.05	6.18
*OsBAHD50*	LOC_Os07g04970.1	Chr7	–	2,187,378	2,188,939	476	50.57	6.38
*OsBAHD51*	LOC_Os08g01950.1	Chr8	–	594,453	598,041	480	51.72	6.41
*OsBAHD52*	LOC_Os08g01960.1	Chr8	–	610,522	612,123	534	57.58	6.26
*OsBAHD53*	LOC_Os08g01980.1	Chr8	–	632,775	634,169	465	49.87	6.02
*OsBAHD54*	LOC_Os08g02020.1	Chr8	–	652,657	654,279	485	51.73	7.61
*OsBAHD55*	LOC_Os08g02030.1	Chr8	–	655,748	657,391	469	49.56	6.12
*OsBAHD56*	LOC_Os08g07720.1	Chr8	–	4,316,160	4,318,638	483	50.13	5.03
*OsBAHD57*	LOC_Os08g07730.1	Chr8	+	4,325,887	4,329,768	470	48.78	5.95
*OsBAHD58*	LOC_Os08g10420.1	Chr8	–	6,121,818	6,123,641	459	50.24	8.56
*OsBAHD59*	LOC_Os08g44840.1	Chr8	–	28,162,970	28,165,431	446	47.62	6.58
*OsBAHD60*	LOC_Os09g25460.1	Chr9	–	15,251,886	15,256,328	441	47.47	5.82
*OsBAHD61*	LOC_Os09g37180.1	Chr9	+	21,450,580	21,452,648	438	47.38	6.23
*OsBAHD62*	LOC_Os09g37200.1	Chr9	+	21,463,619	21,465,476	453	48.62	6.42
*OsBAHD63*	LOC_Os10g01660.1	Chr10	–	435,411	438,047	465	50.73	6.74
*OsBAHD64*	LOC_Os10g01680.1	Chr10	+	448,670	452,960	497	54.77	7.73
*OsBAHD65*	LOC_Os10g01690.1	Chr10	+	455,987	457,291	435	47.71	8.34
*OsBAHD66*	LOC_Os10g01720.1	Chr10	+	468,565	472,415	509	55.9	7.83
*OsBAHD67*	LOC_Os10g01800.1	Chr10	+	526,147	527,331	395	42.9	4.59
*OsBAHD68*	LOC_Os10g01920.1	Chr10	+	583,024	584,677	435	47.01	5.96
*OsBAHD69*	LOC_Os10g01930.1	Chr10	+	591,184	592,703	448	48.69	5.75
*OsBAHD70*	LOC_Os10g02000.1	Chr10	+	632158	633,300	381	41.76	8.58
*OsBAHD71*	LOC_Os10g03360.1	Chr10	+	1,431,142	1,432,407	422	45.3	7.01
*OsBAHD72*	LOC_Os10g03390.1	Chr10	+	1,444,002	1,445,321	423	45.47	6.59
*OsBAHD73*	LOC_Os10g11980.1	Chr10	+	6,639,765	6,645,325	486	52.22	9.03
*OsBAHD74*	LOC_Os10g18430.1	Chr10	+	9,367,214	9,368,185	150	16.19	4.47
*OsBAHD75*	LOC_Os10g23310.1	Chr10	–	12,181,085	12,183,127	437	46.73	5.78
*OsBAHD76*	LOC_Os10g23820.1	Chr10	+	12,214,879	12,216,579	435	47.11	5.41
*OsBAHD77*	LOC_Os10g23820.2	Chr10	+	12,214,985	12,216,523	312	34.08	5.56
*OsBAHD78*	LOC_Os10g26290.1	Chr10	–	13,611,824	13,614,202	457	47.59	5.97
*OsBAHD79*	LOC_Os11g07960.1	Chr11	–	4,128,529	4,130,571	448	48.54	7.89
*OsBAHD80*	LOC_Os11g13970.1	Chr11	–	7,745,980	7,747,416	479	51.01	6.83
*OsBAHD81*	LOC_Os11g31090.1	Chr11	+	18,092,965	18,095,638	463	49.32	4.8
*OsBAHD82*	LOC_Os11g42290.1	Chr11	–	25,466,557	25,468,131	446	47.72	6.51
*OsBAHD83*	LOC_Os11g42370.1	Chr11	+	25,501,586	25,503,169	449	48.03	6.38
*OsBAHD84*	LOC_Os11g42480.1	Chr11	–	25,583,964	25,585,370	443	47.7	5.02
*OsBAHD85*	LOC_Os12g04080.1	Chr12	–	1,713,136	1,714,665	447	48.07	6.97

^a^ aa, amino acid residue.

### 2.4 Phylogenetic and sequence analysis

The complete protein sequences of identified *BAHD‐AT* gene family members of rice, *Arabidopsis*, soybean, sorghum, maize, and *B. distachyon* were used to determine their evolutionary relationship in different plant species. The multiple sequence alignment of complete protein sequences was performed through ClustalW2 with default parameters, and the phylogenetic tree was constructed using MEGA v6.06 (https://www.megasoftware.net). Tree topology was inferred by the neighbor‐joining method with 1,000 bootstrap replicates.

### 2.5 Plant material and inoculation with *R. solani* AG1‐IA

Two rice genotypes, that is, a germplasm line designated as PAU‐ShB8, a moderately resistant genotype (hereinafter referred to as ShB) and PR114, a susceptible genotype (hereinafter referred to as PR) with varying levels of susceptibility to *R. solani* AG1‐IA (Aggarwal et al., 2019), were grown in pots containing sterile soil mix. The fungal inoculum was prepared by placing a single sclerotium (compact mass of hardened mycelium) of *R. solani* AG1‐IA on a potato dextrose agar (HiMedia) plate and incubating it at 28 °C under dark conditions until the plate was full of fungal sclerotia (∼4 d). The 7–8‐week‐old rice plants were inoculated at the second top‐most collar of the tiller by placing a single sclerotium on the inner side of the sheath (Ghosh et al., 2017). The inoculated plants were maintained at 28 °C temperature, 80% relative humidity, and 16/8 h light/dark photoperiod inside a growth chamber (Conviron). The sheath samples containing both the healthy and lesion contacting part ∼1 cm up and down the site of inoculation were collected at 0, 24, 48, and 72 h postinoculation (hpi). The samples were collected in three biological replicates for each genotype, where a replicate consisted of a pooled sheath from four to five tillers of a rice plant, immediately frozen in liquid nitrogen, and stored at −80 °C until further use.

### 2.6 RNA‐seq analysis

The 100‐mg tissue samples were used for total RNA isolation using the RNeasy Plant Mini Kit (Qiagen) as per the manufacturer's instructions. To remove the DNA contamination, an on‐column DNase treatment was given to the isolated total RNA. The NanoDrop UV‐VIS spectrophotometer and denaturing formaldehyde agarose gel were used to determine the concentration and the integrity of isolated RNA, respectively. The RNA‐seq libraries for all time intervals were prepared using TruSeq RNA sample preparation kit v2 (Illumina Inc.) following the manufacturer′s instructions. The library quantification and the insert size determination were performed using Qubit 2.0 fluorometer (Life Technologies) and Agilent Bioanalyzer DNA 1000 chip (Agilent Technologies), respectively. The prepared libraries were sequenced for paired‐end (2 by 100) sequencing using NovaSeq 6000 system (Illumina Inc.) at Council of Scientific and Industrial Research–Institute of Himalayan Bioresource Technology, Palampur. The raw reads were filtered using Trimmomatic v 2.39 (Bolger et al., 2014), and the filtered high‐quality reads were then mapped to the rice reference genome (https://rapdb.dna.affrc.go.jp). The raw reads data is submitted to NCBI Sequence Read Archive (SRA) as a BioProject with accession number PRJNA702874. The data were analyzed for differential gene expression using TopHat and Cufflinks tools (Trapnell et al., 2012). The value of transcript abundance was presented as fragments per kilobase of exon model per million mapped reads (FPKM). The log_2_ fold change in the FPKM values was employed for the heat map construction with hierarchical clustering by using the MeV v4.4.1 software package (http://mev.tm4.org).

### 2.7 qRT‐PCR analysis

The 1.0 μg of total RNA was converted to complementary DNA (cDNA) using the Verso cDNA synthesis kit as per manufacturer instructions (Thermo Fisher Scientific). The cDNA was 10‐fold diluted before performing the quantitative real‐time polymerase chain reaction (qRT‐PCR) assays in a 20‐μl reaction using SYBR green PCR master mix (Thermo Fisher Scientific, USA), on a real‐time PCR thermocycler (Agilent, USA). Each qRT‐PCR assay was performed with three independent biological replicates, each consisting of three independent technical replicates. The transcript abundance of ß‐tubulin was used to normalize the qRT‐PCR data before calculating the relative fold‐change employing the delta‐delta C_t_ (cycle threshold) method (Livak & Schmittgen, 2001). The primer sequences used in the present study are listed in Supplemental Table S1.

### 2.8 Bisulfite sequencing and analysis

The genomic DNA was isolated using GenElute Plant Genomic DNA Miniprep Kit (Sigma‐Aldrich) and fragmented to 200–300 bp via sonication using Adaptive Focused Acoustics (Covaris). The fragmented DNA was then ligated to HiSeq‐methylated adapters before bisulfite treatment (DNA Methylation‐Gold kit, Zymo Research Corporation). The sequencing of bisulfite‐treated DNA was performed on the Illumina HiSeq platform in paired‐end mode (2 by 150 bp), with >30× sequencing depth of the analyzed samples to the rice genome. A total of eight samples consisting of control and *R. solani* AG1‐IA infected samples (24, 48, and 72 hpi) of both genotypes were used for whole‐genome bisulfite sequencing. Each sample was a pool of three independent biological replicates. The adaptor sequences and the low‐quality raw reads were processed and removed using trim galore v0.4.1, with default parameters (https://www.ncbi.nlm.nih.gov/pmc/articles/PMC3532080/). The raw reads data is submitted to NCBI Sequence Read Archive as a BioProject with accession number PRJNA692031. The high‐quality reads were mapped on the rice reference genome (v7, retrieved from http://rice.plantbiology.msu.edu) and aligned using the BatMeth2 package (https://bmcbioinformatics.biomedcentral.com/articles/10.1186/s12859‐018‐2593‐4). To determine the methylation level at each methylcytosine site, we calculated the percentage of reads with methylation call (C) to the total reads aligned (C and T) at the same site.

## 3 RESULTS AND DISCUSSION

### 3.1 Identification and annotation of *BAHD‐AT* gene family members in different plant species

The ‘hmmsearch’ for the transferase domain (PF02458.15) resulted in the identification of a total of 145 rice proteins. Among the identified 145 proteins, the pfamscan, with 1 × 10^−4^ e‐value cutoff, confirmed the presence of transferase domain in only 92 rice proteins. Subsequently, the 92 candidate proteins were then subjected to ‘find individual motif occurrences’ search, with 1 × 10^−3^ e‐value cutoff, to identify the signature motifs, that is, HXXXDG and DFGWG, which reduced the total putative BAHD‐ATs to 85. Parallelly, the BLASTp search of already characterized BAHD‐AT proteins against the rice protein dataset resulted in the identification of 86 putative rice BAHD‐AT proteins, where the subsequent pfamscan showed the absence of BAHD transferase domain in one protein and thus excluded from the analysis. By combining the results of ‘hmmsearch’ program and BLASTp, we were able to identify a total of 85 rice BAHD‐AT proteins encoded by 85 rice gene models. The rice *BAHD‐AT* gene annotations reported in the public databases (NCBI and UniProt) are highly disordered; therefore, we assigned a uniform nomenclature to the 85 rice *BAHD‐AT* genes. The rice BAHD‐AT proteins were designated as *OsBAHD*, followed by Arabic numbers 1–85 according to the position of their encoding genes on linkage groups (LGs) 1–12 in 5′ to 3′orientation. Similar criteria of nomenclature have been employed for the NAC proteins in potato (*Solanum tuberosum* L.) (Singh et al., 2013), HyPRP proteins in rice (Kapoor et al., 2019), and MADS‐box proteins in apple (*Malus domestica* (Suckow) Borkh.) (G. Kumar et al., 2016a). The average length of identified rice OsBAHD proteins was ∼450 amino acids that range from 150 to 623 amino acids (Table 1). The average molecular weight of OsBAHD proteins was 48.4 kDa, where the lowest and the highest molecular weights were 16.19 and 66.67 kDa, respectively (Table 1). In addition to rice, we also identified BAHD‐ATs in two dicot plants (*Arabidopsis* and soybean) and three monocot plants (Sorghum, maize, and *B. distachyon*) using a similar approach. A total of 41, 102, 83, 75, and 74 *BAHD‐AT* encoding genes were identified in *Arabidopsis*, soybean, sorghum, maize, and *B. distachyon*, respectively. The list of *BAHD‐AT* genes identified in these plant species, along with their characteristics, is provided as Supplemental Table S2.

### 3.2 *OsBAHD‐AT* chromosomal distribution, duplication events, structural analysis, and subcellular localization of encoded proteins

**FIGURE 1:**
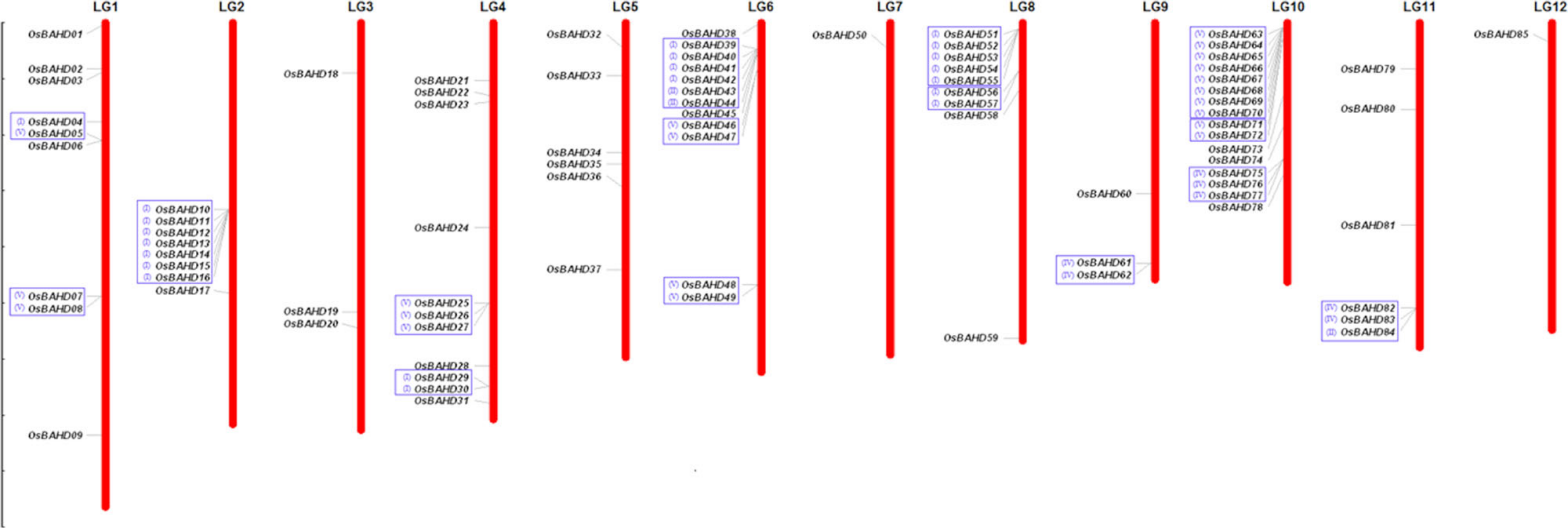
Chromosomal mapping and the clustering of 85 *OsBAHD‐AT* encoding genes on 12 rice linkage groups (LGs). The genes shown in boxes indicate their presence within the 200 Kbp region to form a gene cluster. The clade to which genes present in the cluster belongs is shown in parenthesis

The genomic coordinates for genes encoding 85 *OsBAHD‐ATs* were used to map them on 12 rice LGs. The 85 members of the *OsBAHD‐AT* gene family showed non‐random distribution on 12 rice LGs, where more than 76% of *OsBAHD‐AT* genes were found to be localized on six LGs (LG1, LG2, LG4, LG6, LG8, and LG10) with the highest of 16 *OsBAHD‐AT* genes on LG10 (Figure 1). Often, the clustered distribution on the chromosomes is observed for the genes that belong to a family. The clustering of *OsBAHD‐AT* genes showed 15 clusters comprised of a total of 51 *OsBAHD‐AT* genes distributed on eight rice LGs (Figure 1). The LG2 and LG10 contain the largest clusters comprised of seven and eight genes, respectively. Additionally, the LG6 and LG10 have a maximum number of three clusters, each with 10 and 13 genes, respectively, while LG1, LG2, LG4, LG9, and LG11 have either one or two gene clusters.

The analysis of the rice complete genome sequence has revealed the ancient whole‐genome duplication and recent segmental duplication in the rice genome (Yu et al., 2005). From the duplication analysis of the rice genome, we identified 12,127 (17.89%) genes as tandemly duplicated and 5,654 (8.34%) genes as segmental duplicated. These duplication events in the rice genome may also lead to the expansion of the *OsBAHD‐AT* gene family in rice. Among all the *OsBAHD‐AT* genes, 10 were located on the segmentally duplicated regions of LG1, LG2, LG4, LG5, and LG8 (Supplemental Figure S1). The three *OsBAHD‐AT* pairs were present in duplicated segments on each LG1 and LG5, followed by two *OsBAHD‐AT* genes on LG4 and one each on LG2 and LG8. Additionally, 23 *OsBAHD‐AT* genes were found to be tandemly duplicated (Supplemental Figure S2). Therefore, these results suggested that the duplication events in the rice genome have contributed to the expansion of the *OsBAHD‐AT* gene family. Results from the gene structure analysis of *OsBAHD‐AT* genes showed that the majority of them (36 genes, 42%) have either single intron (37 genes, 43.5%) or intronless (36 genes, 42.3%), while the remaining genes (12 genes, 14%) have introns that vary from two to three in number (Supplemental Figure S3).

The OsBAHD‐ATs were further predicted for their localization in the different subcellular components such as chloroplast, cytoplasm, and plasma membrane using CELLO and WoLF PSORT (Supplemental Table S3). According to CELLO prediction, OsBAHD‐ATs scored relatively higher for their localization in the chloroplast, whereas WoLF PSORT predicted *OsBAHD‐AT* gene localization mainly in the cytoplasm. Several OsBAHD‐ATs are potentially localized in different subcellular compartments and also showed multiorganellar localization in Golgi bodies and plastids (dual), cytoplasm, and nucleus (dual), chloroplast, nucleus, mitochondria, peroxisome, and vacuoles, etc. In total, the differences in the prediction of OsBAHD‐ATs were notable between the two prediction programs (Supplemental Table S3). To date, no prior information is known about the characterization of molecular function and subcellular localization of OsBAHD‐ATs in rice. These results indicate the possible function of BAHD‐ATs in different organelles including the cytosol. These findings were concurrent with the findings of Yu et al. (2009) and Tuominen et al. (2011) in *Populus* and *Arabidopsis*. The prediction of BAHD‐ATs in different subcellular compartments apart from the cytosol suggests the diverse functionality of BAHD‐ATs in the biosynthesis and modification of a repertoire of metabolites, primarily the plants' secondary and specialized metabolites (Peng et al., 2016). Similarly, Bontpart et al. (2015) stated that the concomitant activities of different BAHD‐ATs in different compartments may synergistically promote the accumulation of the metabolites. Also, acylated defense molecules synthesized in the cytosols are possibly toxic to the plant, so vacuolar and multicellular localization and trafficking activities may offer an alternate means of coping with this scenario at various stages of growth, tissues, and environmental conditions.

### 3.3 Structural and phylogenetic analysis of OsBAHD proteins

The multiple sequence alignment of *OsBAHD‐AT* genes along with the representative BAHD‐ATs of *Arabidopsis* (AAN09797; AT3G03480.1 and NP_199704; AT5G48930.1), tobacco (*Nicotiana tabacum L*.) (BAD93691 and CAE46932), apple (AAU14879), maize (CAA61258), opium poppy (*Papaver somniferum* L.) (AAK73661), barley (*Hordeum vulgare* L.) (AAO73071), Concord grape (*Vitis labrusca* L.) (AAW22989), lupin (*Lupinus albus* L.) (BAD89275), and Canadian yew (*Taxus canadensis* Marshall) (AAM75818) revealed that most of the OsBAHD‐ATs contain highly conserved N‐terminal transferase domain with conserved HXXXDG motif and a less conserved C‐terminal DFGWG motif as described previously (D'Auria, 2006) (Supplemental Figure S4).

**FIGURE 2:**
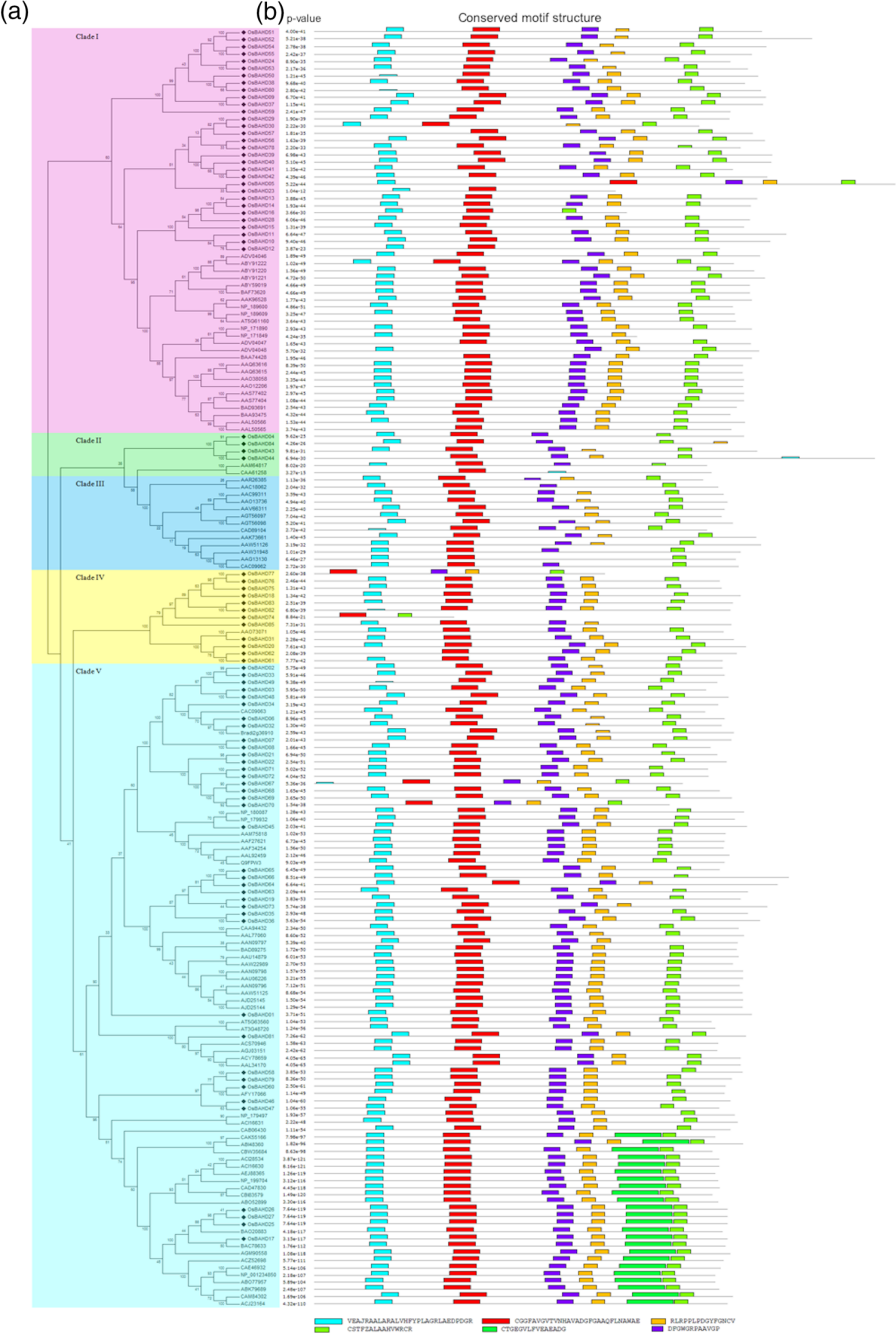
Phylogenetic relationship and the conserved motif composition of *OsBAHD‐ATs*. (A) The phylogenetic tree of *OsBAHD‐AT* proteins and biochemically characterized BAHD‐AT proteins of other plants. The multiple sequence alignment and the phylogenetic tree construction were carried out using MEGA v6.06 using the maximum likelihood method with 1,000 bootstrap replicates. The tree topology revealed the five classical phylogenetic clades (I to V). (B) The MEME analysis revealed the conserved motifs in *OsBAHD‐ATs* and their schematic representation. Each motif shown here is represented by a colored box with a consensus given at the bottom

The OsBAHD‐ATs sequences were used to generate the phylogenetic relationship with the characterized BAHD ‐ATs from other plant species (Bontpart et al., 2015; D'Auria, 2006). The phylogenetic analysis helped us to classify the OsBAHD‐ATs in five different clades from I to V (Figure 2). This classification may indicate the possible functional similarity of OsBAHD‐ATs with that of characterized BAHD‐ATs. Most of the OsBAHD‐ATs were grouped in clade V with 38 OsBAHD‐AT members, followed by 31 members to clade I, 12 members to clade IV, and four members were classified in clade II (Figure 2). The clade III of the characterized BAHD‐ATs contains none of the BAHD‐AT members from rice and other monocots, and this suggests the clade III members are dicot specific and may have some exclusive function in dicot species. Interestingly, clade IV, which contains only one characterized BAHD‐AT from barley (AA073071), has 12 members of OsBAHD‐ATs with significant sequence similarity as determined through high bootstrap value (Figure 2).

**FIGURE 3:**
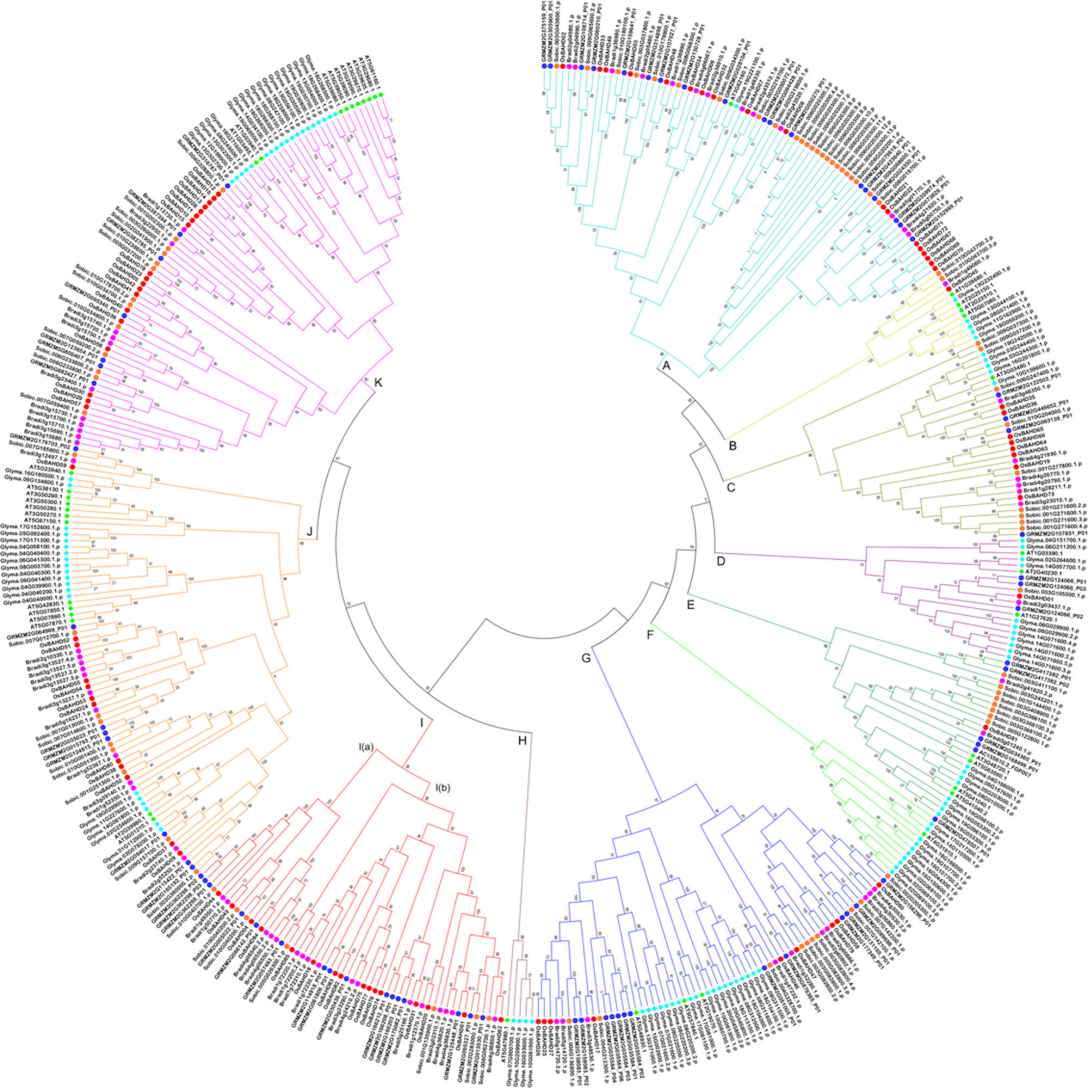
Phylogenetic relationship of OsBAHD‐ATs with that of *Arabidopsis*, *G. max*, *Z. mays*, *S. bicolor*, and *B. distachyon*. The amino acid sequence of mature BAHD‐AT proteins was used for sequence alignment and subsequent construction of the phylogenetic tree employing MEGA v6.06. The different shapes with color codes are given to the BAHDs of different plant species. The sphere indicates the monocots, while the diamond indicates the dicots. The color codes in different shapes are as follows: red for rice, orange for *S. bicolor*, blue for *Z. mays*, magenta for *B. distachyon*, lawn‐green for *Arabidopsis*, and cyan for *G. max*. The tree was divided into 11 major clades named sequentially from A to K

To analyze the phylogenetic relationship of rice BAHD‐ATs with that of other closely related monocots (Sorghum, maize, and *B. distachyon*) and some distant dicots (*Arabidopsis* and soybean) species, we constructed an unrooted phylogenetic tree for the OsBAHD‐ATs and BAHD‐AT proteins of other plant species. The phylogenetic tree classifies 85 OsBAHD‐ATs into 11 distinct phylogenetic clades (A to K) along with their orthologs in other monocot and dicot plant species (Figure 3). Although the BAHD‐ATs were found to be distributed uniformly among the major clades, it is apparent that the BAHD‐ATs of monocots and dicots clustered into distinct subclades of major phylogenetic clades. Exceptionally, clade I contains only monocot‐specific BAHD‐ATs, while clade H contains only the dicot‐specific BAHD‐ATs (Figure 3). The subclade I(b) contains BAHD‐AT members that belong to the clade IV of the phylogenetic tree containing characterized BAHD‐AT members (Figures 2 and 3). Interestingly, I subclade contains none of the dicot BAHD‐AT members, and this observation indicates that subclade I specifically belong to the monocot species (Figure 3).

Among the genetically and biochemically characterized BAHD‐ATs (D'Auria, 2006), there is no apparent specificity for the acyl ‐donors and ‐acceptors among the members of a given clade. Broadly, the members of clade I use malonyl‐CoA as an acyl donor to acylate the phenolic compounds. Similarly, clade V members mainly utilize benzoate‐CoA and hydroxycinnamoyl‐CoA to acylate various metabolites including terpenoids, alkaloids, and medium‐chain aliphatic and aromatic esters (D'Auria, 2006) (Bontpart et al., 2015). However, the hydroxycinnamoyl‐CoA has been reported as an acyl donor for members of multiple clades (D'Auria, 2006). Mutagenesis coupled crystallographic structure analysis of two different BAHD‐ATs revealed the structural diversity among the binding sites for acyl‐donor and ‐acceptor molecules (Unno et al., 2007). It also provides plausible cause for the utilization of vast diversity of acceptor molecules and relatively limited acyl‐donors by different BAHD‐ATs, thus, a potential challenge for phylogeny‐based inference on the functional similarity of the other BAHD‐AT family members.

### 3.4 Expression profiling of *OsBAHD‐AT* genes in response to *R. solani* AG1‐IA identified several putative candidate genes

**FIGURE 4:**
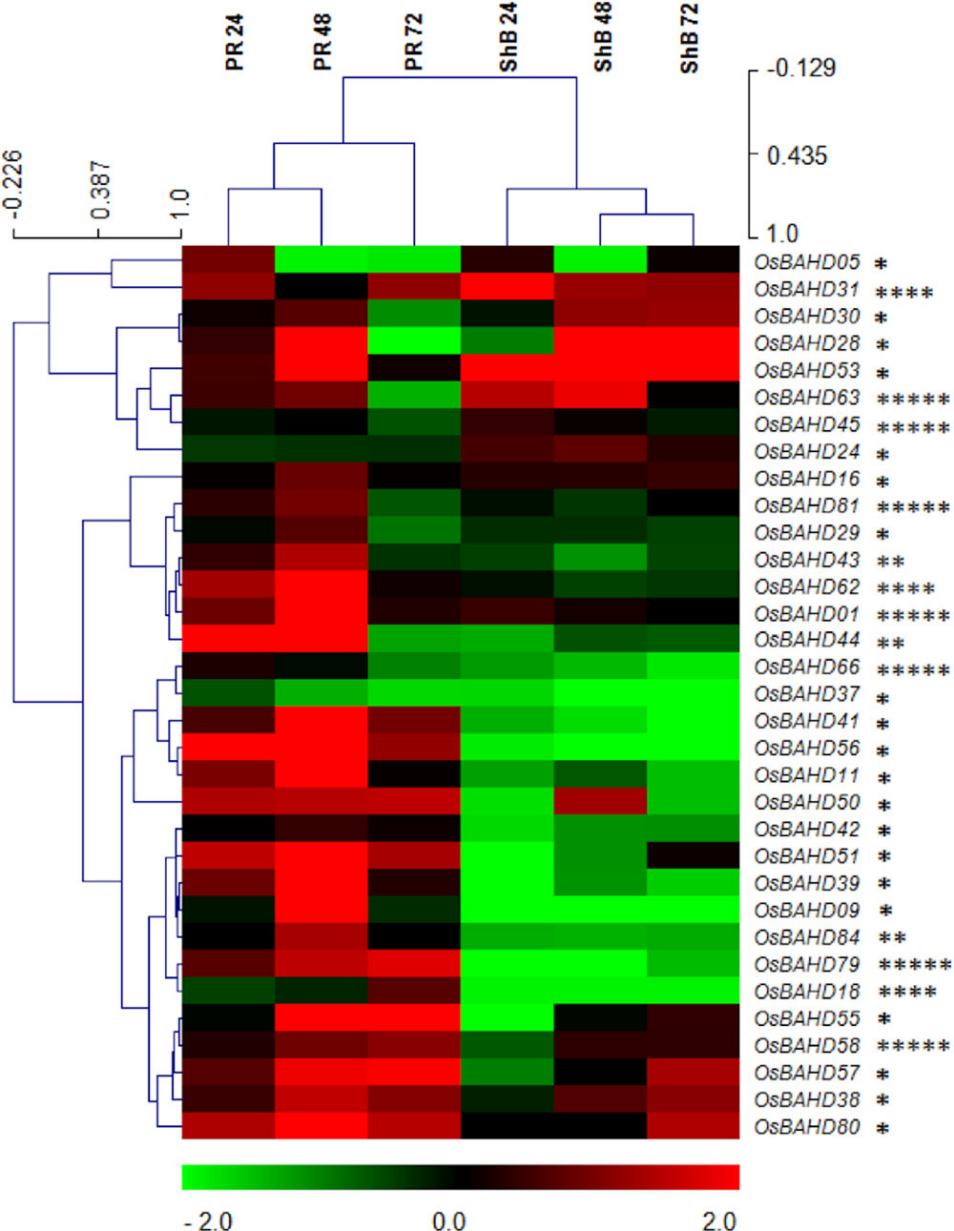
The heat map representation for the transcript abundance level of *OsBAHD‐AT* genes in *R. solani* AG1‐IA challenged rice genotypes viz. PR and ShB at different time intervals. The number of an asterisk (*) next to the gene name indicates the clade to which a particular gene belongs

**FIGURE 5:**
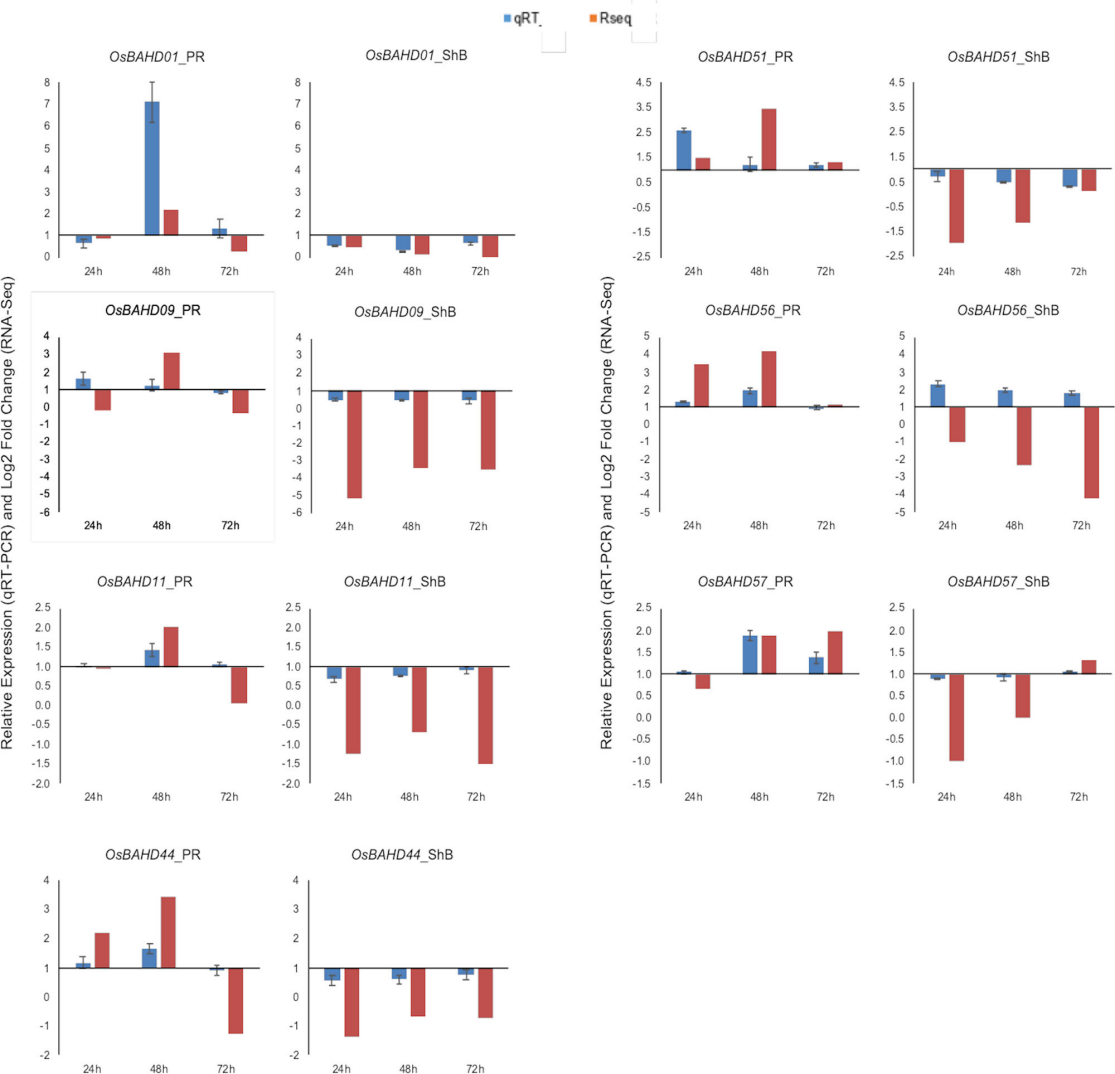
The qualitative real‐time PCR (qRT‐PCR)‐based expression of selected *OsBAHD‐AT* genes in *R. solani* AG1‐IA inoculated rice genotypes. All the qRT‐PCR assays were performed with three biological and their respective technical replicates. Error bars represent the standard error in the replicates

The tissue type has a profound effect on gene expression, therefore our expression experiment had the tissue type from the rice sheath where the fungus inoculation and the infection was established. This strategy helped to identify the localized as well the global response of the fungus infection. The log_2_ fold‐change in the *OsBAHD‐AT* genes expression vs. 0 hpi is given as a heatmap (Figure 4). Out of a total of 85 *OsBAHD‐AT* genes, 33 exhibited expression in all the samples collected after *R. solani* AG1‐IA inoculation. The RNA‐seq data suggest that the expression of *OsBAHD‐AT* gene family members in germplasm line ShB was mostly downregulated or remained unchanged upon *R. solani* AG1‐IA inoculation. In contrast, 29 out of 33 *OsBAHD‐AT* genes were upregulated and only four were downregulated at one or other time‐point after inoculation in susceptible variety PR. However, the genes, namely *OsBAHD28*, *OsBAHD53* of clade I, and *OsBAHD63* of clade V, exhibited upregulated expression in ShB during the later stages of postinoculation. Interestingly, the *OsBAHD‐AT* genes exhibiting dysregulation of expression (change in expression vs. control) mostly belonged to clade I, and the majority of them (12 out of 20 genes) were either downregulated or had reduced expression in ShB upon *R. solani* AG1‐IA inoculation. Importantly, in contrast, these genes of clade I exhibited higher expression in susceptible genotype PR. The *OsBAHD38*, *OsBAHD57*, and *OsBAHD80* genes had higher expression in ShB after *R. solani* AG1‐IA inoculation; however, the expression of these genes was comparatively more in PR. This data suggests the importance of clade I *OsBAHD‐AT* genes in rice sheath blight disease. Also, the genes of clade IV, namely *OsBAHD18* and *OsBAHD62*, except *OsBAHD31*, had lower expression in the susceptible vs. the resistant genotype. To validate the RNA‐seq‐based expression profiling, the qRT‐PCR assay was conducted for the randomly selected genes, that is, *OsBAHD01*, *OsBAHD09*, *OsBAHD11*, *OsBAHD44*, *OsBAHD51*, *OsBAHD56*, *and OsBAHD57* in the *R. solani* AG1‐IA challenged samples of ShB and PR. Since qRT‐PCR and RNA‐seq data of the selected genes shows a common pattern of expression in the respective rice genotypes, the qRT‐PCR data corroborate the RNA‐seq data (Figure 5).

In one of the previous studies, the expression of the *OsBAHD57* was found to be upregulated by 3.7‐ and 2.8‐fold in two susceptible rice genotypes (PB1 and TP309, respectively) challenged with *R. solani* AG1‐IA (Ghosh et al., 2018). In the present study, the *OsBAHD57* has similar upregulated expression (>1.9‐fold) in the susceptible genotype PR upon fungal inoculation, while its expression remains unchanged in resistant genotype ShB (Figures 4 and 5). Altogether, it can be concluded that the *OsBAHD‐AT* gene family has an important role in rice–*R. solani* AG1‐IA interaction. The BAHD‐ATs acylate the flavonoids and the other secondary metabolites to diversify their biological role in mitigating the environmental stresses (Bontpart et al., 2015), the higher expression of *BAHD‐AT* gene family members in susceptible rice genotype PR suggests their negative role in rice sheath‐blight resistance (Figure 4). One of the previous studies reported that a mutant of a novel member of the *BAHD‐AT* gene family, *eps1* in *Arabidopsis*, had compromised resistance against *Pseudomonas syringae* because of reduced pathogen‐induced salicylic acid (SA) accumulation and expression of pathogenesis‐related genes (Zheng et al., 2009); where the exogenous SA application restored the disease resistance that suggests EPS1 works upstream of SA. However, it was also observed that *Arabidopsis* overexpressing *ABS1*, another novel *BAHD‐AT*, has the resemblance of brassinosteroid (BR) deficient or signaling mutant with dwarf phenotype, which can be rescued by BR treatment, thus state the function of *ABS1* upstream to BR (M. Wang et al., 2012). Likewise, the BRASSINOSTEROID INSENSITIVE 1 (BIA1) and BIA2 BAHD‐AT‐like proteins were found to be involved in the inactivation of BR to maintain the BR homeostasis in *Arabidopsis* (Gan et al., 2020; Roh et al., 2012; Zhang & Xu, 2018). These studies thus established the link between BAHD‐ATs and BR biosynthesis and signaling. The exogenous application of brassinolide (a most common and important BR) in tobacco and rice was observed to enhance the disease resistance in tobacco and rice against biotrophic fungi and suggested that the BR‐mediated disease resistance works independently of the systemic acquired resistance (Nakashita et al., 2003). It is also evident that an exogenous application of BRs has protective effects against a broad range of plant pathogens including fungi, bacteria, and viruses (Bajguz & Hayat, 2009). In a challenge to this prevailing view that BRs positively regulated the disease resistance in rice, a study showed that rice pathogen *Pythium graminicola* used BRs as a virulence factor to infect rice possibly mediated through negative cross‐talk with SA and gibberellic acid pathways (De Vleesschauwer et al., 2012). Similarly, it was recently found that rice *BRI1* mutant *d61‐1* (BR signaling mutant) and the mutant of BR biosynthesis gene *d2* are highly resistant to *R. solani* (Yuan et al., 2018). Though these pieces of evidence are conflicting to define the role of BR in rice sheath blight resistance, the detailed study in *Arabidopsis* has demonstrated both the antagonistic and synergistic effect of BR in plant's innate immunity and is a function of BR homeostasis (Albrecht et al., 2012; Belkhadir et al., 2012). Additionally, it was suggested that the variation in the plant disease resistance response through pattern‐triggered immunity depends on the relative levels of BR and its receptor proteins BRI1 and BAK1 (BRI1 associated kinase 1) (Wang, 2012). This relative wealth of information on BAHD‐ATs and BRs suggests that the upregulation of *OsBAHD‐AT* genes in PR may perturb the BR homeostasis and eventually BR‐mediated disease resistance, thus explain the plausible enhanced susceptibility to sheath‐blight disease in PR. However, conclusive experimental evidences on how exogenous BR treatment affect the BAHD expression and eventually disease tolerance are required to validate this hypothesis.

### 3.5 Methylation status of *OsBAHD‐ATs* in *R. solani* AG1‐IA challenged rice

**FIGURE 6:**
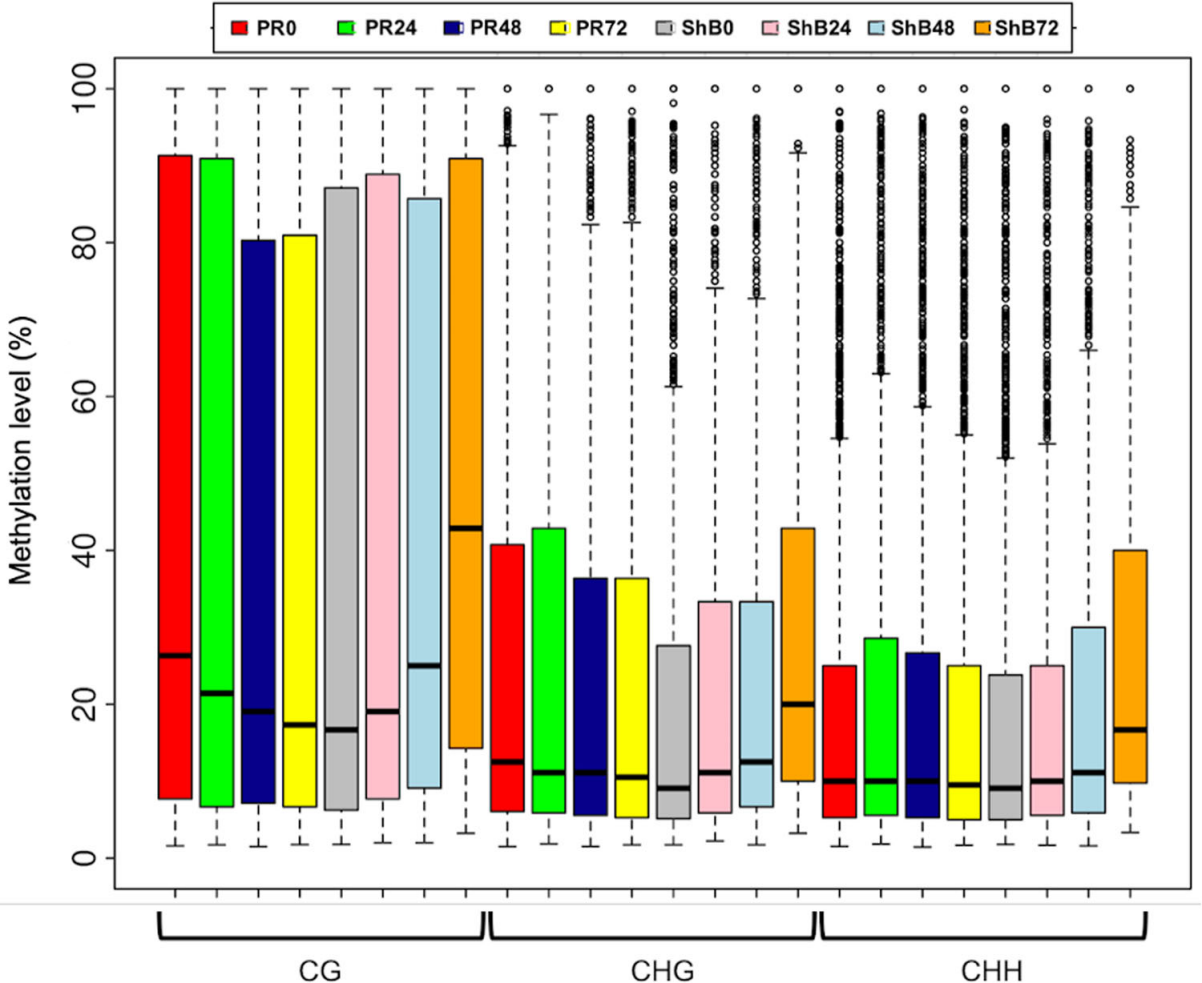
Change in the methylation level at individual methylated cytosine site of *OsBAHD‐ATs* in different sequence contexts, in the control and *R. solani* AG1‐IA inoculated rice genotypes, is shown via box plot. Each box shows the interquartile range for the number of methylated cytosines

Epigenetic modification is a well‐known strategy used by plants to adapt to various environmental changes without changing the genetic sequence. DNA cytosine methylation is an epigenetic modification that affects gene expression and is thus associated with transcriptional regulation in various plants (Gallego‐Bartolomé, 2020; G. Kumar et al., 2016b). Therefore, in the present study, we analyzed the dynamics of cytosine DNA methylation in the *OsBAHD* genes and their 2‐kb flanking regions at three sequence contexts, that is, CG, CHG, and CHH. The analysis of the methylation level of *OsBAHD‐AT* genes showed a higher percentage of methylated cytosine in the CG context, followed by CHG and CHH in all the analyzed samples of both genotypes (Figure 6). Interestingly, the percentage of methylated cytosines of *OsBAHD‐AT* genes in resistant genotype ShB gradually increased after fungal inoculation in all the sequence contexts (CG, CHG, and CHH). In contrast, the overall reduction in methylated cytosines was observed in susceptible genotype PR.

**FIGURE 7:**
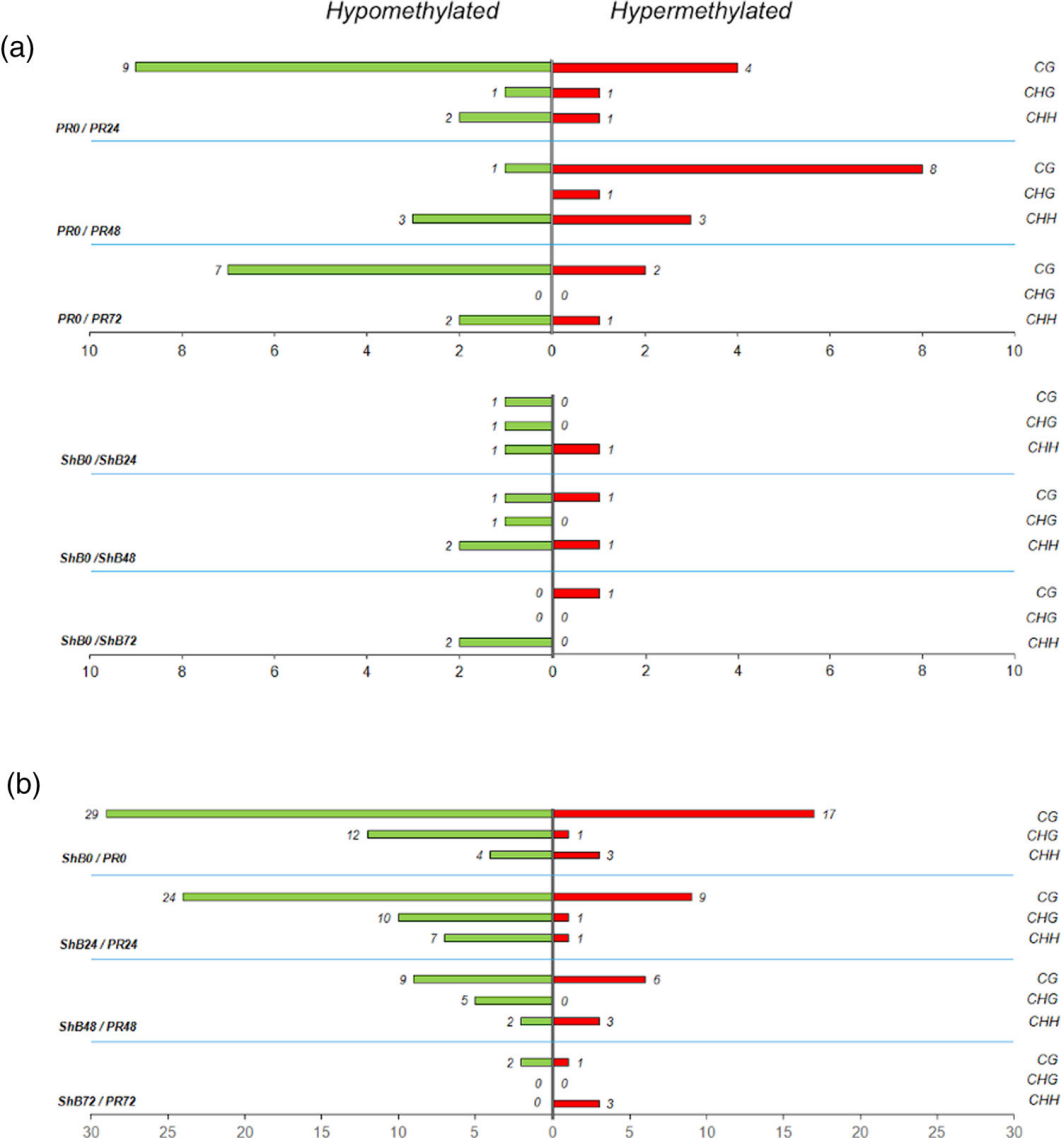
Differentially methylated regions (DMRs) of *OsBAHD‐ATs*. (A) Intra‐genotype DMRs considering uninoculated samples as control. (B) Intergenotype comparison of DMRs in corresponding samples, considering ShB as control, upon *R. solani* AG1‐IA inoculation

The analysis of differential methylated regions (DMRs) of *OsBAHD‐AT* genes showed more hypomethylation at all the time points postinfection (24, 48, and 72 hpi) in PR, whereas in ShB the number of hypo‐ and hypermethylated regions remained nearly constant (Figure 7). In the comparison between genotypes, ShB vs. PR, the higher number of DMRs were hypomethylated in PR genotype at corresponding time points as compared with hypermethylated regions. The reduction in total methylation level (Figure 6) and hypomethylation of the *OsBAHD‐AT* genes (Figure 7) may explain their corresponding upregulated and higher expression in the PR genotype (Figure 5). Though no previous reports are available on epigenetic modification of *BAHD‐AT* genes, from our results it may be inferred that the expression of OsBAHD‐ATs encoding genes is epigenetically regulated in *R. solani* challenged sheath blight susceptible genotype PR.

## 4 CONCLUSIONS

The present study identified and characterized the *BAHD‐AT* gene family comprised of 85 members. The genome organization of *OsBAHD‐AT* gene members showed nonrandom distribution and localized as clusters on all 12 rice LGs. The duplication analysis showed that the expansion of the rice *BAHD‐AT* gene family has mostly occurred through tandem duplication. The phylogenetic analysis of *OsBAHD‐AT* genes along with other characterized BAHD‐AT proteins of other plant species enabled us to classify the OsBAHD‐ATs in five distinct clades, where none of *OsBAHD‐AT* members belongs to clade III. The high‐throughput RNA‐seq analysis allowed us to determine the expression of *OsBAHD‐AT* genes, where the expression of 33 members was detected in *R. solani* AG1‐IA challenged rice genotypes having differential tolerance to sheath blight disease. The changes in cytosine methylation level and DMR indicates the epigenetic regulation of rice *BAHD‐AT* gene expression in response to *R. solani*. The population‐wide DMR studies for *BAHD* gene loci may develop the confidence of using *OsBAHD‐AT* genes as epiallele for rice crop improvement strategies. We also discussed the plausible link in the *OsBAHD‐AT* gene expression, BR homeostasis, and the sheath blight resistance; however, further experimental validation is required. This systematic study of *OsBAHD‐AT* genes provided a foundation for future gene functional characterization and their potential applications in rice genetic improvement for sheath blight resistance. Altogether the present study indicated an importance of *OsBAHD‐AT* genes to devise strategies for the development of sheath blight disease‐resistant rice cultivars.

## AUTHOR CONTRIBUTIONS

Gulshan Kumar: Conceptualization; Data curation; Formal analysis; Investigation; Methodology; Software; Validation; Visualization; Writing‐original draft; Writing‐review & editing. Pankaj Kumar: Formal analysis; Investigation; Methodology; Validation. Ritu Kapoor: Formal analysis; Investigation; Software. Jagjeet Singh Lore: Methodology; Resources. Dharminder Bhatia: Methodology; Resources. Arun Kumar: Conceptualization; Data curation; Formal analysis; Funding acquisition; Investigation; Methodology; Project administration; Resources; Supervision; Validation; Visualization; Writing‐original draft; Writing‐review & editing.

## CONFLICT OF INTEREST

The authors declare no conflict of interest.

## Supporting information


**Supplemental Figure S1**. Collinear pairs of *OsBAHD‐AT* genes on all 12 rice chromosomes.

**Supplemental Figure S2**. Tandem and collinear pairs of duplicated *OsBAHD‐AT* genes as predicted through MCScanX.

**Supplemental Figure S3**. The multiple sequence alignment for protein sequences of rice BAHD‐ATs and characterized BAHD‐ATs from other plants.

**Supplemental Table S1**: List of primers used for RT‐qPCR expression validation analysis.

**Supplemental Table S2**. List of *BAHD‐AT* gene models identified from different monocot and dicot plant species for comparative phylogenetic analysis.

**Supplemental Table S3**. Prediction of subcellular localization of the rice OsBAHD family proteins.
