# Supplementary material for: Characterization of evolutionarily distinct rice BAHD‐Acyltransferases provides insight into their plausible role in rice susceptibility to Rhizoctonia solani
Source: Plant Genome. 2021 Sep 9;14(3):e20140. doi: 10.1002/tpg2.20140 (PMC12807277; doi:10.1002/tpg2.20140)
Supplement: Supplementary file 1 [file TPG2-14-e20140-s004.docx]

**Supplementary Table S1:** List of primers used for RT-qPCR expression validation analysis.

| **Name** | **MSU locus ID** | **RAPDB locus ID** | **Forward primer (5'→3')** | **Reverse primer (5'→3')** |
| --- | --- | --- | --- | --- |
| *OsBAHD01* | LOC_Os01g01520.1 | Os01g0104900 | CGACACCTTCGAGCACCAT | TGTCGTGGTCCTGCTTGTC |
| *OsBAHD09* | LOC_Os01g63480.1 | Os01g0854000 | CCTCTTTGGCCTGAATTGCG | CCGATCGATTGGCGTTTGTG |
| *OsBAHD11* | LOC_Os02g28200.1 | Os02g0483800 | TGCACGACCACGTAAAGACT | CTTTCCTTGATGCTCTGCAG |
| *OsBAHD44* | LOC_Os06g05790.1 | Os06g0151100 | CCATCCAGTTGCCGTACGA | TCGACGAGCAGGTGGTTGGT |
| *OsBAHD51* | LOC_Os08g01950.1 | Os08g0111300 | CGTCAACCATCTCGTCGCT | ACCGTGTAGAACGTCACCTG |
| *OsBAHD56* | LOC_Os08g07720.1 | Os08g0174100 | CGCGTGTTCCTGTACGAGAT | CACGTAGACCAGCTTCCCTG |
| *OsBAHD57* | LOC_Os08g07730.1 | Os08g0174300 | TCGGTCTGGAGGTTCATCGA | TACTTCCGGAGCACGTC |
