# Supplementary material for: Characterization of evolutionarily distinct rice BAHD‐Acyltransferases provides insight into their plausible role in rice susceptibility to Rhizoctonia solani
Source: Plant Genome. 2021 Sep 9;14(3):e20140. doi: 10.1002/tpg2.20140 (PMC12807277; doi:10.1002/tpg2.20140)
Supplement: Supplementary file 2 [file TPG2-14-e20140-s005.docx]

**Supplementary Table S2.** List of *BAHD-AT* gene models identified from different monocot and dicot plant species for comparative phylogenetic analysis.

| **Dicots** | | | **Monocots** | | |
| --- | --- | --- | --- | --- | --- |
|  | **Length (aa)** | **kDa** |  | **Length (aa)** | **kDa** |
| ***Arabidopsis*** | | | ***B. distachyon*** | | |
| AT1G03390.1 | 461 | 52.09 | Bradi1g12370.1.p | 464 | 50.01 |
| AT1G03495.1 | 465 | 51.41 | Bradi1g13730.1.p | 471 | 49.37 |
| AT1G03940.1 | 469 | 51.93 | Bradi1g28211.1.p | 458 | 50.5 |
| AT1G27620.1 | 442 | 49.81 | Bradi1g32280.1.p | 455 | 48.71 |
| AT1G28680.1 | 451 | 49.69 | Bradi1g36980.1.p | 433 | 46.71 |
| AT2G19070.1 | 451 | 50.44 | Bradi1g36990.1.p | 455 | 49.01 |
| AT2G23510.1 | 451 | 50.38 | Bradi1g47232.1.p | 475 | 51.13 |
| AT2G25150.1 | 461 | 51.76 | Bradi1g49060.1.p | 474 | 50.82 |
| AT2G39980.1 | 482 | 52.89 | Bradi1g49330.1.p | 432 | 45.36 |
| AT2G40230.1 | 433 | 47.67 | Bradi1g49350.1.p | 476 | 50.82 |
| AT3G03480.1 | 454 | 50.3 | Bradi1g50770.2.p | 473 | 49.42 |
| AT3G29590.1 | 449 | 49.44 | Bradi1g52350.1.p | 473 | 50.61 |
| AT3G29635.1 | 458 | 51.16 | Bradi1g52367.1.p | 458 | 48.85 |
| AT3G29670.1 | 451 | 50.22 | Bradi1g72200.2.p | 451 | 48.89 |
| AT3G29680.1 | 451 | 50.73 | Bradi1g72210.1.p | 443 | 48.02 |
| AT3G48720.1 | 430 | 48.01 | Bradi1g72220.1.p | 447 | 48.21 |
| AT3G50270.1 | 450 | 49.81 | Bradi1g72230.1.p | 438 | 47.49 |
| AT3G50280.1 | 443 | 49.97 | Bradi2g03437.1.p | 489 | 51.95 |
| AT3G50290.1 | 273 | 30.75 | Bradi2g04980.1.p | 438 | 46.66 |
| AT3G50300.1 | 448 | 50.25 | Bradi2g04990.1.p | 439 | 47.02 |
| AT3G62160.1 | 428 | 47.56 | Bradi2g05480.1.p | 446 | 48.58 |
| AT4G31910.1 | 458 | 51.15 | Bradi2g10330.1.p | 470 | 50.52 |
| AT5G01210.1 | 475 | 52.34 | Bradi2g23740.1.p | 495 | 52.83 |
| AT5G07080.1 | 450 | 48.96 | Bradi2g36910.1.p | 450 | 48.78 |
| AT5G07850.1 | 456 | 50.76 | Bradi2g41620.2.p | 427 | 45.37 |
| AT5G07860.1 | 454 | 50.44 | Bradi2g43510.1.p | 427 | 45.43 |
| AT5G07870.1 | 464 | 51.75 | Bradi2g43520.1.p | 424 | 45.75 |
| AT5G23940.1 | 484 | 53.52 | Bradi2g55250.1.p | 505 | 53.55 |
| AT5G38130.1 | 462 | 52.1 | Bradi2g60886.1.p | 436 | 47.43 |
| AT5G39050.1 | 469 | 51.77 | Bradi2g60892.1.p | 437 | 47.44 |
| AT5G39080.1 | 463 | 51.49 | Bradi3g02310.1.p | 470 | 51.44 |
| AT5G39090.1 | 448 | 49.92 | Bradi3g12497.1.p | 451 | 47.95 |
| AT5G41040.1 | 457 | 50.96 | Bradi3g13237.1.p | 466 | 50.34 |
| AT5G41040.2 | 441 | 49.26 | Bradi3g13527.2.p | 527 | 57.3 |
| AT5G42830.1 | 450 | 50.53 | Bradi3g13527.3.p | 527 | 57.27 |
| AT5G47980.1 | 443 | 48.53 | Bradi3g13527.4.p | 466 | 50.19 |
| AT5G48930.1 | 433 | 47.98 | Bradi3g13527.5.p | 466 | 50.19 |
| AT5G57840.1 | 443 | 49.96 | Bradi3g15680.1.p | 451 | 47.9 |
| AT5G61160.1 | 452 | 51.08 | Bradi3g15690.1.p | 457 | 48.15 |
| AT5G63560.1 | 426 | 47.12 | Bradi3g15700.1.p | 445 | 47.36 |
| AT5G67150.1 | 448 | 49.94 | Bradi3g15710.1.p | 458 | 48.2 |
|  |  |  | Bradi3g15720.1.p | 490 | 52.14 |
| ***G. max*** | | | Bradi3g15730.1.p | 455 | 47.82 |
| Glyma.01G112900.1.p | 475 | 52.05 | Bradi3g15740.1.p | 475 | 50.8 |
| Glyma.02G069100.1.p | 457 | 50.97 | Bradi3g15750.1.p | 467 | 49.61 |
| Glyma.02G069100.2.p | 334 | 37.12 | Bradi3g22950.1.p | 462 | 48.98 |
| Glyma.02G069100.3.p | 317 | 35.23 | Bradi3g23010.1.p | 470 | 51.7 |
| Glyma.02G254600.1.p | 479 | 53.4 | Bradi3g23280.1.p | 433 | 47.13 |
| Glyma.02G264600.1.p | 441 | 48.65 | Bradi3g36860.1.p | 440 | 47.55 |
| Glyma.03G078200.1.p | 468 | 51.23 | Bradi3g39140.1.p | 477 | 50.57 |
| Glyma.03G244300.1.p | 465 | 51.77 | Bradi3g46350.1.p | 454 | 48.97 |
| Glyma.03G244400.1.p | 476 | 52.88 | Bradi3g48530.1.p | 443 | 48.35 |
| Glyma.04G039900.1.p | 462 | 52.05 | Bradi4g06067.1.p | 443 | 47.77 |
| Glyma.04G040000.1.p | 406 | 45.28 | Bradi4g06540.2.p | 478 | 51.84 |
| Glyma.04G040200.1.p | 470 | 52.52 | Bradi4g06550.1.p | 493 | 53.38 |
| Glyma.04G040300.1.p | 487 | 55.02 | Bradi4g06550.2.p | 493 | 53.38 |
| Glyma.04G040400.1.p | 461 | 51.68 | Bradi4g20770.1.p | 457 | 50.45 |
| Glyma.04G058100.1.p | 461 | 51.7 | Bradi4g20790.1.p | 454 | 50.28 |
| Glyma.04G151700.1.p | 461 | 51.76 | Bradi4g21920.1.p | 462 | 49.03 |
| Glyma.04G198000.1.p | 432 | 47.99 | Bradi4g21930.1.p | 453 | 49.92 |
| Glyma.05G092400.1.p | 437 | 48.5 | Bradi4g24210.2.p | 396 | 43.33 |
| Glyma.05G203000.1.p | 434 | 48.36 | Bradi4g30530.1.p | 434 | 46.98 |
| Glyma.06G029900.1.p | 453 | 51.1 | Bradi4g30530.2.p | 317 | 34.86 |
| Glyma.06G029900.2.p | 344 | 38.81 | Bradi4g36820.1.p | 436 | 47.66 |
| Glyma.06G041300.1.p | 458 | 51.35 | Bradi4g36830.1.p | 437 | 47.89 |
| Glyma.06G041400.1.p | 457 | 51.31 | Bradi4g36850.1.p | 444 | 48.68 |
| Glyma.06G167600.1.p | 432 | 47.94 | Bradi5g00750.1.p | 467 | 48.74 |
| Glyma.06G211200.1.p | 462 | 51.72 | Bradi5g01240.1.p | 456 | 48.74 |
| Glyma.07G000700.1.p | 425 | 47.65 | Bradi5g01770.1.p | 387 | 40.68 |
| Glyma.07G021600.1.p | 466 | 51.62 | Bradi5g14720.1.p | 444 | 48.47 |
| Glyma.07G021600.2.p | 439 | 48.42 | Bradi5g14720.2.p | 444 | 48.47 |
| Glyma.08G003700.1.p | 306 | 34.36 | Bradi5g16237.1.p | 485 | 52.08 |
| Glyma.08G010500.1.p | 431 | 47.83 | Bradi5g23400.1.p | 449 | 48.82 |
| Glyma.08G071400.1.p | 473 | 52.77 | Bradi5g25166.1.p | 452 | 49.29 |
| Glyma.08G220200.1.p | 430 | 47.51 |  |  |  |
| Glyma.08G220200.2.p | 430 | 47.51 | ***S. bicolor*** | | |
| Glyma.08G220200.3.p | 430 | 47.51 | Sobic.001G002300.1.p | 480 | 50.97 |
| Glyma.08G247100.1.p | 448 | 49.42 | Sobic.001G135900.1.p | 459 | 50.02 |
| Glyma.08G249500.1.p | 470 | 52.32 | Sobic.001G251300.1.p | 472 | 50.63 |
| Glyma.08G311500.1.p | 466 | 52.34 | Sobic.001G271600.1.p | 470 | 52.05 |
| Glyma.08G311600.1.p | 477 | 53.9 | Sobic.001G271600.2.p | 470 | 52.34 |
| Glyma.08G312000.1.p | 453 | 50.66 | Sobic.001G271600.3.p | 450 | 50.16 |
| Glyma.09G134600.1.p | 449 | 49.74 | Sobic.001G271600.4.p | 419 | 46.39 |
| Glyma.10G060700.1.p | 449 | 50.32 | Sobic.001G277800.1.p | 462 | 49.93 |
| Glyma.10G061300.1.p | 431 | 48.16 | Sobic.002G041900.1.p | 481 | 51.26 |
| Glyma.10G061900.1.p | 467 | 52.08 | Sobic.002G205300.1.p | 434 | 47.26 |
| Glyma.10G159600.1.p | 460 | 50.51 | Sobic.002G283000.1.p | 444 | 47.98 |
| Glyma.10G209900.1.p | 374 | 41.87 | Sobic.003G037800.1.p | 418 | 46.29 |
| Glyma.11G162900.1.p | 399 | 44 | Sobic.003G043600.1.p | 445 | 47.24 |
| Glyma.11G227600.1.p | 494 | 54.22 | Sobic.003G082800.1.p | 416 | 45.45 |
| Glyma.12G199900.1.p | 476 | 53.38 | Sobic.003G082800.2.p | 416 | 45.45 |
| Glyma.13G044100.1.p | 463 | 52.05 | Sobic.003G082800.3.p | 392 | 42.87 |
| Glyma.13G054000.1.p | 470 | 51.32 | Sobic.003G082800.4.p | 392 | 42.87 |
| Glyma.13G056100.1.p | 468 | 51.67 | Sobic.003G105500.1.p | 498 | 53.55 |
| Glyma.13G232400.1.p | 453 | 50.42 | Sobic.003G219700.1.p | 429 | 45.48 |
| Glyma.13G302300.1.p | 470 | 52.89 | Sobic.003G219800.1.p | 427 | 45.83 |
| Glyma.13G302700.1.p | 469 | 52.59 | Sobic.003G245201.1.p | 691 | 73.45 |
| Glyma.13G371000.1.p | 440 | 48.79 | Sobic.003G260000.1.p | 486 | 51.73 |
| Glyma.14G034100.1.p | 474 | 54.21 | Sobic.003G360000.1.p | 496 | 53.24 |
| Glyma.14G057700.1.p | 442 | 48.66 | Sobic.003G368100.1.p | 456 | 48.84 |
| Glyma.14G061800.1.p | 480 | 53.49 | Sobic.003G368100.2.p | 457 | 48.97 |
| Glyma.14G071600.1.p | 449 | 50.4 | Sobic.003G368100.3.p | 456 | 48.84 |
| Glyma.14G071600.2.p | 449 | 50.4 | Sobic.003G408900.1.p | 434 | 46.18 |
| Glyma.14G071600.3.p | 341 | 38.14 | Sobic.003G411100.1.p | 446 | 48.96 |
| Glyma.14G071600.4.p | 341 | 38.14 | Sobic.004G212300.1.p | 449 | 48.84 |
| Glyma.14G071600.5.p | 341 | 38.14 | Sobic.004G328700.1.p | 452 | 48.11 |
| Glyma.14G110500.1.p | 479 | 53.18 | Sobic.004G328800.1.p | 469 | 49.75 |
| Glyma.15G002600.1.p | 375 | 41.34 | Sobic.005G026300.1.p | 454 | 48.01 |
| Glyma.16G033600.1.p | 456 | 50.85 | Sobic.005G037200.1.p | 459 | 49.06 |
| Glyma.16G044300.1.p | 448 | 49.88 | Sobic.005G122800.1.p | 465 | 50.13 |
| Glyma.16G053300.1.p | 454 | 50.82 | Sobic.006G019700.1.p | 479 | 51.16 |
| Glyma.16G053300.2.p | 435 | 48.39 | Sobic.006G020200.1.p | 427 | 45.47 |
| Glyma.16G150600.1.p | 458 | 51.15 | Sobic.006G020300.10.p | 429 | 45.96 |
| Glyma.16G180500.1.p | 450 | 49.96 | Sobic.006G020300.11.p | 311 | 33.55 |
| Glyma.16G201800.1.p | 456 | 50.65 | Sobic.006G020300.12.p | 311 | 33.55 |
| Glyma.17G061000.1.p | 461 | 51.27 | Sobic.006G020300.13.p | 311 | 33.55 |
| Glyma.17G061100.1.p | 456 | 50.69 | Sobic.006G020300.2.p | 429 | 45.96 |
| Glyma.17G152600.1.p | 444 | 49.05 | Sobic.006G020300.3.p | 429 | 45.96 |
| Glyma.17G171300.1.p | 440 | 49.44 | Sobic.006G020300.4.p | 429 | 45.96 |
| Glyma.17G217200.1.p | 480 | 53.29 | Sobic.006G020300.5.p | 429 | 45.96 |
| Glyma.18G029900.1.p | 474 | 51.71 | Sobic.006G020300.6.p | 429 | 45.96 |
| Glyma.18G056200.1.p | 461 | 51.01 | Sobic.006G020300.7.p | 429 | 45.96 |
| Glyma.18G104000.1.p | 467 | 52.72 | Sobic.006G020300.8.p | 429 | 45.96 |
| Glyma.18G104100.1.p | 463 | 52.23 | Sobic.006G020300.9.p | 429 | 45.96 |
| Glyma.18G113100.1.p | 481 | 54.49 | Sobic.006G062700.1.p | 459 | 49.96 |
| Glyma.18G166000.1.p | 442 | 49.05 | Sobic.006G136800.1.p | 442 | 48.28 |
| Glyma.18G258000.1.p | 512 | 56.72 | Sobic.006G233800.1.p | 457 | 49.34 |
| Glyma.18G268100.1.p | 480 | 52.92 | Sobic.006G233800.2.p | 457 | 49.34 |
| Glyma.18G268200.1.p | 477 | 52.95 | Sobic.006G247400.1.p | 452 | 49.09 |
| Glyma.18G268300.1.p | 478 | 52.64 | Sobic.007G012700.1.p | 486 | 52.19 |
| Glyma.18G268400.1.p | 451 | 50.19 | Sobic.007G013000.1.p | 464 | 50.04 |
| Glyma.18G268500.1.p | 451 | 49.92 | Sobic.007G014600.1.p | 474 | 50.68 |
| Glyma.18G271600.1.p | 371 | 41.83 | Sobic.007G059200.2.p | 503 | 53 |
| Glyma.19G030500.1.p | 464 | 51.23 | Sobic.007G059400.1.p | 479 | 49.83 |
| Glyma.19G030700.1.p | 477 | 52.81 | Sobic.007G142100.1.p | 444 | 48.18 |
| Glyma.19G030800.1.p | 465 | 51.19 | Sobic.007G142200.1.p | 447 | 47.51 |
| Glyma.19G069500.1.p | 479 | 54.13 | Sobic.007G144400.1.p | 359 | 37.87 |
| Glyma.19G096100.1.p | 433 | 48.01 | Sobic.007G165800.1.p | 468 | 49.52 |
| Glyma.19G096100.2.p | 433 | 48.01 | Sobic.008G068300.1.p | 441 | 48.36 |
| Glyma.19G107700.1.p | 445 | 49.63 | Sobic.009G034300.1.p | 437 | 47.22 |
| Glyma.19G107700.2.p | 422 | 47.12 | Sobic.009G037200.1.p | 471 | 49.9 |
| Glyma.19G221100.1.p | 433 | 47.66 | Sobic.009G037300.1.p | 472 | 50.72 |
| Glyma.19G242000.1.p | 465 | 51.87 | Sobic.009G065600.2.p | 442 | 47.55 |
| Glyma.20G045800.1.p | 465 | 52.63 | Sobic.009G068600.1.p | 450 | 47.83 |
| Glyma.20G045800.2.p | 462 | 52.36 | Sobic.009G157100.1.p | 516 | 54.05 |
|  |  |  | Sobic.010G001300.1.p | 494 | 53.26 |
|  |  |  | Sobic.010G001400.1.p | 462 | 50.28 |
|  |  |  | Sobic.010G034700.1.p | 454 | 48.76 |
|  |  |  | Sobic.010G034800.1.p | 478 | 50.54 |
|  |  |  | Sobic.010G040200.1.p | 460 | 48.86 |
|  |  |  | Sobic.010G040200.2.p | 460 | 48.86 |
|  |  |  | Sobic.010G040700.1.p | 477 | 51.13 |
|  |  |  | Sobic.010G043700.2.p | 457 | 48.98 |
|  |  |  | Sobic.010G043700.3.p | 457 | 48.98 |
|  |  |  | Sobic.010G178700.2.p | 481 | 50.66 |
|  |  |  | Sobic.010G179900.1.p | 447 | 48.29 |
|  |  |  | Sobic.010G180100.1.p | 439 | 47.36 |
|  |  |  | Sobic.010G204000.1.p | 486 | 53.35 |
|  |  |  | Sobic.010G238600.1.p | 480 | 50.9 |
|  |  |  |  |  |  |
|  |  |  | ***Z. mays*** | | |
|  |  |  | AC155610.2_FGP007 | 447 | 47.7 |
|  |  |  | GRMZM2G005522_P01 | 460 | 48.78 |
|  |  |  | GRMZM2G013530_P01 | 450 | 48.47 |
|  |  |  | GRMZM2G015793_P01 | 490 | 51.73 |
|  |  |  | GRMZM2G024350_P01 | 446 | 47.38 |
|  |  |  | GRMZM2G028104_P01 | 436 | 47.15 |
|  |  |  | GRMZM2G030436_P01 | 442 | 47.97 |
|  |  |  | GRMZM2G034360_P01 | 454 | 48.56 |
|  |  |  | GRMZM2G035023_P01 | 488 | 51.42 |
|  |  |  | GRMZM2G035584_P01 | 447 | 48.63 |
|  |  |  | GRMZM2G035584_P02 | 447 | 48.63 |
|  |  |  | GRMZM2G035584_P03 | 447 | 48.63 |
|  |  |  | GRMZM2G035584_P04 | 320 | 34.64 |
|  |  |  | GRMZM2G035584_P06 | 447 | 48.63 |
|  |  |  | GRMZM2G050072_P01 | 429 | 45.49 |
|  |  |  | GRMZM2G050270_P01 | 429 | 46.1 |
|  |  |  | GRMZM2G051005_P01 | 443 | 47.25 |
|  |  |  | GRMZM2G057483_P01 | 457 | 48.39 |
|  |  |  | GRMZM2G060210_P01 | 431 | 46.66 |
|  |  |  | GRMZM2G061806_P01 | 444 | 47.78 |
|  |  |  | GRMZM2G063139_P01 | 480 | 52.46 |
|  |  |  | GRMZM2G064969_P01 | 481 | 52.14 |
|  |  |  | GRMZM2G066142_P01 | 463 | 49.7 |
|  |  |  | GRMZM2G073829_P01 | 442 | 46.95 |
|  |  |  | GRMZM2G079616_P01 | 426 | 45.34 |
|  |  |  | GRMZM2G089698_P01 | 477 | 51.28 |
|  |  |  | GRMZM2G094017_P01 | 543 | 57.36 |
|  |  |  | GRMZM2G094428_P01 | 429 | 45.95 |
|  |  |  | GRMZM2G095327_P01 | 446 | 48.06 |
|  |  |  | GRMZM2G095340_P01 | 466 | 50.49 |
|  |  |  | GRMZM2G107027_P01 | 479 | 51.84 |
|  |  |  | GRMZM2G107851_P01 | 471 | 52.66 |
|  |  |  | GRMZM2G108714_P01 | 437 | 47.34 |
|  |  |  | GRMZM2G114918_P01 | 444 | 47.71 |
|  |  |  | GRMZM2G115422_P01 | 511 | 54.39 |
|  |  |  | GRMZM2G122503_P01 | 479 | 51.85 |
|  |  |  | GRMZM2G123654_P01 | 486 | 50.79 |
|  |  |  | GRMZM2G124066_P01 | 476 | 51.21 |
|  |  |  | GRMZM2G124066_P02 | 317 | 33.89 |
|  |  |  | GRMZM2G124066_P03 | 183 | 19.75 |
|  |  |  | GRMZM2G124815_P01 | 476 | 51.59 |
|  |  |  | GRMZM2G125448_P01 | 445 | 48.36 |
|  |  |  | GRMZM2G130728_P01 | 434 | 47.74 |
|  |  |  | GRMZM2G131165_P01 | 432 | 46.8 |
|  |  |  | GRMZM2G152969_P01 | 441 | 47.75 |
|  |  |  | GRMZM2G156296_P01 | 436 | 46.61 |
|  |  |  | GRMZM2G158083_P01 | 441 | 48.28 |
|  |  |  | GRMZM2G158083_P02 | 441 | 48.28 |
|  |  |  | GRMZM2G159641_P01 | 426 | 45.66 |
|  |  |  | GRMZM2G165192_P01 | 492 | 52.8 |
|  |  |  | GRMZM2G166203_P01 | 456 | 49.27 |
|  |  |  | GRMZM2G166208_P01 | 465 | 50.83 |
|  |  |  | GRMZM2G166212_P01 | 463 | 50.2 |
|  |  |  | GRMZM2G168499_P01 | 282 | 30.93 |
|  |  |  | GRMZM2G175082_P01 | 468 | 49.55 |
|  |  |  | GRMZM2G177349_P01 | 427 | 46.45 |
|  |  |  | GRMZM2G179703_P02 | 445 | 47.59 |
|  |  |  | GRMZM2G305900_P01 | 457 | 48.43 |
|  |  |  | GRMZM2G314898_P01 | 418 | 46.14 |
|  |  |  | GRMZM2G316787_P01 | 483 | 51.27 |
|  |  |  | GRMZM2G359974_P01 | 591 | 62.32 |
|  |  |  | GRMZM2G362298_P01 | 472 | 50.67 |
|  |  |  | GRMZM2G362298_P02 | 472 | 50.67 |
|  |  |  | GRMZM2G362298_P03 | 492 | 53.12 |
|  |  |  | GRMZM2G375159_P01 | 444 | 46.87 |
|  |  |  | GRMZM2G382785_P01 | 473 | 51.11 |
|  |  |  | GRMZM2G387394_P01 | 484 | 51.7 |
|  |  |  | GRMZM2G417382_P01 | 425 | 46.48 |
|  |  |  | GRMZM2G417382_P02 | 392 | 42.69 |
|  |  |  | GRMZM2G423640_P01 | 425 | 44.64 |
|  |  |  | GRMZM2G446652_P01 | 480 | 52.55 |
|  |  |  | GRMZM2G478507_P01 | 325 | 35.72 |
|  |  |  | GRMZM5G800407_P01 | 486 | 50.79 |
|  |  |  | GRMZM5G878365_P01 | 154 | 16.76 |
|  |  |  | GRMZM5G882427_P01 | 480 | 52.19 |
