# Supplementary material for: Characterization of evolutionarily distinct rice BAHD‐Acyltransferases provides insight into their plausible role in rice susceptibility to Rhizoctonia solani
Source: Plant Genome. 2021 Sep 9;14(3):e20140. doi: 10.1002/tpg2.20140 (PMC12807277; doi:10.1002/tpg2.20140)
Supplement: Supplementary file 3 [file TPG2-14-e20140-s002.doc]

**Supplementary Table S3.** Prediction of subcellular localization of the rice OsBAHD family proteins.

| **Name** | **CELLO^c^** | **WoLF PSORT^d^** |
| --- | --- | --- |
| OsBAHD01 | Pla_mem (1.9), extr (0.8), nucl (0.6) | cyto (9), chlo (2), nucl (1), plas (1), E.R._vacu (1) |
| OsBAHD02 | chlo (1.6), mito (1.3), pero (0.7) | chlo (4), plas (3), E.R. (3), cyto (2), mito (1), vacu (1) |
| OsBAHD03 | chlo (2.5), cyto (1.2), mito (0.5) | cyto (7), chlo (4), pero (3) |
| OsBAHD04 | cyto (2.6), periplasmic (0.7) | pero (9), cyto (3.5), cyto_nucl (2.5), chlo (1) |
| OsBAHD05 | Pla_mem (1.3), cyto (1.0), chlo (0.9), mito (0.6) | mito (8), chlo (5), cyto (1) |
| OsBAHD06 | chlo (2.1), cyto (0.9), Pla_mem (0.8), mito (0.6) | chlo (5), mito (4), cyto (3), pero (2) |
| OsBAHD07 | chlo (2.0), Pla_mem (1.1) | E.R. (4.5), E.R._plas (3.5), cyto (3), extra (3), mito (2), plas (1.5) |
| OsBAHD08 | chlo (1.4), cyto (1.3), mito (0.5) | cyto (5), chlo (4), extr (3), mito (2) |
| OsBAHD09 | chlo (1.7), Pla_mem (1.1), mito (0.7) | chlo (14) |
| OsBAHD10 | chlo (2.2), Pla_mem (0.8) | mito (8), chlo (5), cyto (1) |
| OsBAHD11 | Pla_mem (1.1), chlo (1.1), mito (1.0), pero (0.7), cyto (0.5) | cyto (7), chlo (6), mito (1) |
| OsBAHD12 | nucl (2.8), mito (1.1) | chlo (9), mito (4), nucl (1) |
| OsBAHD13 | chlo (1.7), cyto (1.2), pero (0.7), mito (0.5) | chlo (6), mito (6), cyto (1), pero (1) |
| OsBAHD14 | chlo (1.9), cyto (0.9), mito (0.6), pero (0.5) | chlo (11), mito (2), vacu (1) |
| OsBAHD15 | chlo (1.3), cyto (1.0), mito (0.9), pero (0.7) | cyt (12), mito (1), plas (1) |
| OsBAHD16 | chlo (1.5), Pla_mem (1.3), mito (0.8), cyto (0.6) | mito (7), chlo (6), pero (1) |
| OsBAHD17 | chlo (1.3), Pla_mem (1.2), mito (1.0), cyto (0.6) | mito (5), chlo (4), nucl (2), pero (2), cyto (1) |
| OsBAHD18 | chlo (1.2), cyto (1.1), mito (0.7),Pla_mem (0.6), pero (0.5) | pero (11), cyto (2), chlo (1) |
| OsBAHD19 | Pla_mem (1.3), cyto (1.2), pero (0.9) | chlo_mito (6.8), chlo (6), mito (5.5), cyto_mito (3.7), nucl (1), cyto (1) |
| OsBAHD20 | chlo (2.0), Pla_mem (1.0) | cyto (12), nucl (1), plas (1) |
| OsBAHD21 | chlo (2.2), Pla_mem (1.1), pero (0.5) | cyto (9), pero (3), chlo (2) |
| OsBAHD22 | chlo (1.6), pero (0.8), cyto (0.7) | cyto (7), chlo (5), mito (1), cysk () |
| OsBAHD23 | nucl (2.0), chlo (0.9), mito (0.7), Pla_mem (0.6), | chlo (13), mito (1) |
| OsBAHD24 | chlo (1.8), cyto (1.3), mito (0.9) | cyto (10), chlo (2), pero (2) |
| OsBAHD25 | Pla_mem (1.6), chlo (1.5), mito (0.6), cyto (0.5) | mito (8.5), chlo_mito (7.5), chlo (5.5) |
| OsBAHD26 | Pla_mem (1.6), chlo (1.5), mito (0.6), cyto (0.5) | mito (8.5), chlo_mito (7.5), chlo (5.5) |
| OsBAHD27 | Pla_mem (1.6), chlo (1.5), mito (0.6), cyto (0.5) | mito (8.5), chlo_mito (7.5), chlo (5.5) |
| OsBAHD28 | chlo (1.8), cyto (1.1), mito (0.6), Pla_mem (0.5) | cyto (8), chlo (4), mito (2) |
| OsBAHD29 | chlo (1.6), Pla_mem (1.0), cyto (0.9), mito (0.8) | chlo (8), cyto (4), nucl (1), mito (1) |
| OsBAHD30 | cyto (1.8), chlo (1.1), pero (0.6), Pla_mem (0.5), mito (0.5) | cyto (8), chlo (2), pero (2), plas (1), E.R._vacu (1) |
| OsBAHD31 | cyto (1.3), chlo (1.1), Pla_mem (0.5) | cyto (10), chlo (4) |
| OsBAHD32 | chlo (2.1), cyto (1.2), mito (0.7) | cyto (6), chlo (5), mito (3) |
| OsBAHD33 | mito (1.6), chlo (1.1), pero (0.9), Pla_mem (0.7) | cyto (8), E. R. (4), mito (2) |
| OsBAHD34 | mito (1.2), chlo (1.2), cyto (0.9), Pla_mem (0.8) | chlo (7), mito (5), cyto (1), pero (1) |
| OsBAHD35 | mito (2.0), pero (0.8), chlo (0.7), cyto (0.6), Pla_mem (0.6) | pero (9), cyto (3), chlo (2) |
| OsBAHD36 | chlo (1.7), Pla_mem (1.0), mito (0.8), cyto (0.6), pero (0.5) | pero (8), chlo (4), cyto (2) |
| OsBAHD37 | Pla_mem (1.4), mito (0.9), chlo (0.8), extr (0.6), pero (0.6) | chlo (12), mito (2) |
| OsBAHD38 | Pla_mem (1.3), chlo (1.0), mito (1.0) | mito (10), chlo (3), cyto (1) |
| OsBAHD39 | chlo (1.7), nucl (0.8), cyto (0.7), Pla_mem (0.6) | extr (4), vacu (4), chlo (2), cyto (1.5), cyto_nucl (1.5), mito (1), E.R. (1) |
| OsBAHD40 | chlo (1.9), Pla_mem (1.3), cyto (0.7) | mito (9), chlo (5) |
| OsBAHD41 | chlo (1.9), Pla_mem (0.7), mito (0.7), cyto (0.5) | chlo (7), cyto (2.5), E.R. (2.5), cyto_nucl (2), E.R._plas (2), vacu (1) |
| OsBAHD42 | cyto (1.3), chlo (1.1), Pla_mem (0.9), nucl (0.6), mito (0.5) | chlo (5), mito (2), vacu (2), E. R. (2), cyto (1.5), cyto_nucl (1.5), extr (1) |
| OsBAHD43 | Pla_mem (2.2), chlo (1.3), mito (0.6) | pero (10), cyto (3), chlo (1) |
| OsBAHD44 | Pla_mem (1.4), chlo (1.4), cyto (0.5) | chlo (12), cyto (1), pero (1) |
| OsBAHD45 | cyto (1.2), chlo (1.1), Pla_mem (1.0) | cyto (6), chlo (3), pero (3), mito (2) |
| OsBAHD46 | chlo (1.5), pero (1.0), cyto (0.8), Pla_mem (0.7), mito (0.6) | chlo (7), plas (3), E.R. (2), cyto (1), vacu (1) |
| OsBAHD47 | Pla_mem (2.0), chlo (0.8), mito (0.7) | cyto (10), chlo (3), mito (1) |
| OsBAHD48 | chlo (1.7), cyto (0.9), pero (0.7), mito (0.6), Pla_mem (0.5) | cyto (10), pero (3), chlo (1) |
| OsBAHD49 | Pla_mem (1.6), chlo (1.2), mito (0.7), pero (0.6) | chlo (11), nucl (1), mito (1), extra (1) |
| OsBAHD50 | chlo (1.9), Pla_mem (1.0) | mito (8), chlo (6) |
| OsBAHD51 | chlo (3.3) | cyto (7), chlo (4), pero (2), mito (1) |
| OsBAHD52 | chlo (2.6), cyto (0.8), Pla_mem (0.6) | cyto (8), pero (3), chlo (2), nucl (1) |
| OsBAHD53 | chlo (2.6), cyto (1.1), mito (0.5) | cyto (10), mito (2), chlo (1), pero (1) |
| OsBAHD54 | chlo (1.8), Pla_mem (1.3), mito (0.7) cyto (0.5) | cyto (9), chlo (2), mito (2), pero (2) |
| OsBAHD55 | chlo (2.7), Pla_mem (1.1), cyto (0.5) | chlo (7), mito (2), plas (2), nucl (1), vacu (1), E.R. (1) |
| OsBAHD56 | cyto (1.2), chlo (1.1), Pla_mem (0.9) | cyto (9), pero (3), chlo (2) |
| OsBAHD57 | cyto (1.2), Pla_mem (1.1), chlo (0.9) | cyto (8), pero (2), chlo (1), nucl (1), plas (1), E.R._vacu (1) |
| OsBAHD58 | chlo (1.2), mito (1.0), Pla_mem (0.9), cyto (0.7), nucl (0.6) | pero (12), nucl (1), cyto (1) |
| OsBAHD59 | Periplasmic (3.0), cyto (0.8) | chlo (13), mito (1) |
| OsBAHD60 | Pla_mem (1.4), chlo (0.9), cyto (0.8), mito (0.6) | cyto (8), chlo (3), pero (2), plas (1) |
| OsBAHD61 | cyto (1.3), chlo (0.7), Pla_mem (0.7), mito (0.6), ER (0.6) | cyto (8), pero (3), chlo (2), E.R._vacu (1) |
| OsBAHD62 | cyto (1.4), chlo (1.2), mito (0.9), Pla_mem (0.6) | cyto (7), chlo (4), mito (2), nucl (1) |
| OsBAHD63 | Pla_mem (2.3), chlo (1.1), mito (0.5), cyto (0.5) | pero (9), cyto (2.5), chlo (2), cyto_nucl (2) |
| OsBAHD64 | Pla_mem (1.7), cyto (1.0), mito (0.7), chlo (0.6) | pero (8), chlo (3), cyto (2), golgi (1) |
| OsBAHD65 | Pla_mem (2.7), chlo (0.8), mito (0.5) | chlo (11), mito (3) |
| OsBAHD66 | Pla_mem (2.7), mito (0.8), chlo (0.7) | mito (7),chlo (6), nucl (1) |
| OsBAHD67 | Pla_mem (1.9), chlo (1.2), cyto (0.7), | cyto (8), chlo (3), pero (2), cysk_nucl (1) |
| OsBAHD68 | chlo (1.4), cyto (1.3), Pla_mem (0.9) | cyto (10), mito (3), chlo (1) |
| OsBAHD69 | chlo (1.6), Pla_mem (1.0), cyto (0.7) | chlo (4), mito (4), cyto (2.5), vacu (2), cyto_nucl (2), plas (1) |
| OsBAHD70 | mito (1.7), chlo (1.1), extr (0.6), nucl (0.6) | chlo (8.5), chlo_mito (7.5),mito (5.5) |
| OsBAHD71 | cyto (1.5), chlo (1.3), mito (0.6), pero (0.6), Pla_mem (0.5) | chlo (5), cyto (3), plas (2), vacu (2), nucl (1), extra (1) |
| OsBAHD72 | chlo (1.5), cyto (1.1), Pla_mem (0.9), pero (0.5) | cyto (11), plas (2), chlo (1) |
| OsBAHD73 | chlo (1.3), mito (1.3), cyto (0.7), Pla_mem (0.6) | mito (8),chlo (4), pero (2) |
| OsBAHD74 | cyto (1.7), Pla_mem (1.2), extr (0.8) | chlo (7), cyto (7) |
| OsBAHD75 | chlo (1.3), cyto (1.1), mito (0.8) | cyto (11), mito (2), plas (1) |
| OsBAHD76 | chlo (1.3), cyto (0.8), Pla_mem (0.7) | cyto (12), mito (1), cysk (1) |
| OsBAHD77 | Pla_mem (1.8), chlo (0.9), cyto (0.6), mito (0.5) | cyto (8), chlo (5), nucl (1) |
| OsBAHD78 | chlo (1.7), cyto (1.0), Pla_mem (0.5) | chlo (13), pero (1) |
| OsBAHD79 | Pla_mem (1.9), chlo (1.3), mito (0.7), cyto (0.5), | cyto (9.5), cyto_nucl (6.5), nucl (2.5), plas (1), E.R._vacu (1) |
| OsBAHD80 | chlo (2.4), mito (0.6), cyto (0.5) | mito (9),chlo (5) |
| OsBAHD81 | chlo (1.9), cyto (1.1), Pla_mem (0.6), mito (0.5) | cyto (8), chlo (6) |
| OsBAHD82 | chlo (1.7), cyto (1.1), Pla_mem (0.8) | pero (13), cyto (1) |
| OsBAHD83 | chlo (1.9), cyto (0.9), Pla_mem (0.7) | pero (14) |
| OsBAHD84 | chlo (2.3), Pla_mem (0.9) | cyto (8), chlo (3), pero (2),plas (1) |
| OsBAHD85 | mito (1.2), chlo (0.9), Pla_mem (0.7), cyto (0.6), pero (0.5) | mito (4), cyto (3.5), cyto_nucl (3), vacu (2), nucl (1.5), chlo (1), E.R.(1), golgi (1) |

(c) The subcellular localization prediction program CELLO was used to determine the possible localization sites of the rice OsBAHD family proteins.

(d) The prediction from the subcellular localization program WoLF PSORT. The numbers in parenthesis indicate prior possible protein localization sites of the OsBAHD. Abbreviations of protein localization sites in the dataset are as follows: nucl- nucleus; chlo- chloroplast; cyto- cytosol; cysk- cytoskeleton; plas- plastids; vacu-vacoule; golg- golgi bodies; E.R.- endoplasmic reticulum; extr- extracellular; E.R._vacu-endoplasmic reticulum and vacoule; mito-mitochondria; pero- peroxisomes; and golgi_plas- golgi bodies and plastids; cyto_nucl - cytosol and nucleus; cysk_nucl - cytoskeleton and nucleus; chlo_mito - chloroplast and mitochondria; cyto_mito - cytosol and mitochondria; Pla_mem - Plasma membrane
